# WSB1/2 target chromatin-bound lysine-methylated RelA for proteasomal degradation and NF-κB termination

**DOI:** 10.1093/nar/gkae161

**Published:** 2024-03-07

**Authors:** Jie Zhang, Yuanyuan Yu, Xiuqun Zou, Yaning Du, Qiankun Liang, Mengyao Gong, Yurong He, Junqi Luo, Dandan Wu, Xiaoli Jiang, Matt Sinclair, Emad Tajkhorshid, Hong-Zhuan Chen, Zhaoyuan Hou, Yuejuan Zheng, Lin-Feng Chen, Xiao-Dong Yang

**Affiliations:** Hongqiao Institute of Medicine, Tongren Hospital/Faculty of Basic Medicine, Shanghai Jiaotong University School of Medicine, Shanghai 200025, China; The Research Center for Traditional Chinese Medicine, Shanghai Institute of Infectious Diseases and Biosecurity, Shanghai University of Traditional Chinese Medicine, Shanghai 201203, China; Center for Traditional Chinese Medicine and Immunology Research, School of Integrative Medicine, Shanghai University of Traditional Chinese Medicine, Shanghai 201203, China; Hongqiao Institute of Medicine, Tongren Hospital/Faculty of Basic Medicine, Shanghai Jiaotong University School of Medicine, Shanghai 200025, China; Hongqiao Institute of Medicine, Tongren Hospital/Faculty of Basic Medicine, Shanghai Jiaotong University School of Medicine, Shanghai 200025, China; The Research Center for Traditional Chinese Medicine, Shanghai Institute of Infectious Diseases and Biosecurity, Shanghai University of Traditional Chinese Medicine, Shanghai 201203, China; Center for Traditional Chinese Medicine and Immunology Research, School of Integrative Medicine, Shanghai University of Traditional Chinese Medicine, Shanghai 201203, China; The Research Center for Traditional Chinese Medicine, Shanghai Institute of Infectious Diseases and Biosecurity, Shanghai University of Traditional Chinese Medicine, Shanghai 201203, China; Center for Traditional Chinese Medicine and Immunology Research, School of Integrative Medicine, Shanghai University of Traditional Chinese Medicine, Shanghai 201203, China; The Research Center for Traditional Chinese Medicine, Shanghai Institute of Infectious Diseases and Biosecurity, Shanghai University of Traditional Chinese Medicine, Shanghai 201203, China; Center for Traditional Chinese Medicine and Immunology Research, School of Integrative Medicine, Shanghai University of Traditional Chinese Medicine, Shanghai 201203, China; The Research Center for Traditional Chinese Medicine, Shanghai Institute of Infectious Diseases and Biosecurity, Shanghai University of Traditional Chinese Medicine, Shanghai 201203, China; Center for Traditional Chinese Medicine and Immunology Research, School of Integrative Medicine, Shanghai University of Traditional Chinese Medicine, Shanghai 201203, China; Shanghai Institute of Immunology, and Department of Immunology and Microbiology, Shanghai Jiao Tong University School of Medicine, Shanghai 200025, China; Shanghai Institute of Immunology, and Department of Immunology and Microbiology, Shanghai Jiao Tong University School of Medicine, Shanghai 200025, China; Theoretical and Computational Biophysics Group, NIH Center for Macromolecular Modeling and Visualization, Beckman Institute for Advanced Science and Technology, and Center for Biophysics and Quantitative Biology, University of Illinois at Urbana-Champaign, Urbana, IL 61801, USA; Department of Biochemistry, University of Illinois at Urbana-Champaign, Urbana, IL 61801, USA; Theoretical and Computational Biophysics Group, NIH Center for Macromolecular Modeling and Visualization, Beckman Institute for Advanced Science and Technology, and Center for Biophysics and Quantitative Biology, University of Illinois at Urbana-Champaign, Urbana, IL 61801, USA; Department of Biochemistry, University of Illinois at Urbana-Champaign, Urbana, IL 61801, USA; The Research Center for Traditional Chinese Medicine, Shanghai Institute of Infectious Diseases and Biosecurity, Shanghai University of Traditional Chinese Medicine, Shanghai 201203, China; Shuguang lab of Future Health, Shanghai Frontiers Science Center of TCM Chemical Biology, Shuguang Hospital, Shanghai University of Traditional Chinese Medicine, Shanghai 201203, China; Hongqiao Institute of Medicine, Tongren Hospital/Faculty of Basic Medicine, Shanghai Jiaotong University School of Medicine, Shanghai 200025, China; Linyi University-Shanghai Jiaotong University Joint Institute of Translational Medicine, Linyi University, Shandong 276000, China; The Research Center for Traditional Chinese Medicine, Shanghai Institute of Infectious Diseases and Biosecurity, Shanghai University of Traditional Chinese Medicine, Shanghai 201203, China; Center for Traditional Chinese Medicine and Immunology Research, School of Integrative Medicine, Shanghai University of Traditional Chinese Medicine, Shanghai 201203, China; Department of Biochemistry, University of Illinois at Urbana-Champaign, Urbana, IL 61801, USA; The Research Center for Traditional Chinese Medicine, Shanghai Institute of Infectious Diseases and Biosecurity, Shanghai University of Traditional Chinese Medicine, Shanghai 201203, China; Center for Traditional Chinese Medicine and Immunology Research, School of Integrative Medicine, Shanghai University of Traditional Chinese Medicine, Shanghai 201203, China

## Abstract

Proteasome-mediated degradation of chromatin-bound NF-κB is critical in terminating the transcription of pro-inflammatory genes and can be triggered by Set9-mediated lysine methylation of the RelA subunit. However, the E3 ligase targeting methylated RelA remains unknown. Here, we find that two structurally similar substrate-recognizing components of Cullin-RING E3 ligases, WSB1 and WSB2, can recognize chromatin-bound methylated RelA for polyubiquitination and proteasomal degradation. We showed that WSB1/2 negatively regulated a subset of NF-κB target genes via associating with chromatin where they targeted methylated RelA for ubiquitination, facilitating the termination of NF-κB-dependent transcription. WSB1/2 specifically interacted with methylated lysines (K) 314 and 315 of RelA via their N-terminal WD-40 repeat (WDR) domains, thereby promoting ubiquitination of RelA. Computational modeling further revealed that a conserved aspartic acid (D) at position 158 within the WDR domain of WSB2 coordinates K314/K315 of RelA, with a higher affinity when either of the lysines is methylated. Mutation of D158 abolished WSB2’s ability to bind to and promote ubiquitination of methylated RelA. Together, our study identifies a novel function and the underlying mechanism for WSB1/2 in degrading chromatin-bound methylated RelA and preventing sustained NF-κB activation, providing potential new targets for therapeutic intervention of NF-κB-mediated inflammatory diseases.

## Graphical Abstract

**Graphical Abstract:**
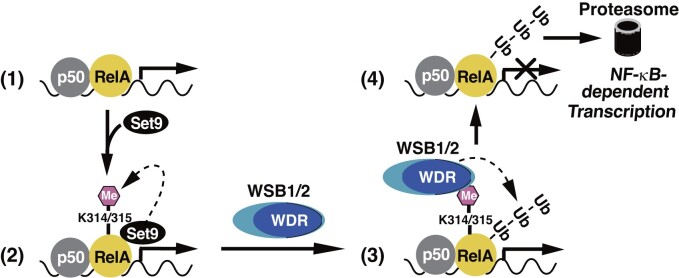

## Introduction

Inflammation is a biological response launched by the immune system to protect against various insults such as infection or injury and is associated with majority of human diseases (1,2). Initiation of an inflammatory response relies on prompt gene transcription that is largely mediated by the nuclear factor-κB (NF-κB) family of transcription factors. NF-κB functions as hetero- or homo-dimers composed of five members of this family, p50, p52, RelA (p65), RelB and c-Rel, in almost all cell types, and the heterodimer RelA/p50 is the most prevalent form in which the RelA subunit has a transactivation domain essential for transcriptional activation of NF-κB target genes (3).

In unstimulated cells, NF-κB is kept in the cytosol in an inactive form by its inhibitor proteins, IκBs (mainly IκBα), but it can be activated by hundreds of distinct ligands, including pathogen-associated molecular patterns (PAMPs) and host cells-secreted cytokines, via dozens of pattern recognition receptors (PRRs) and a large number of cytokine receptors, such as Toll-like receptors and the tumor necrosis factor (TNF) receptors (4). Upon the engagement of these ligands to their cognate receptors, a series of sequential signaling events enable the activation of the inhibitor of κB kinase (IKK) complex, leading to the phosphorylation and degradation of IκB inhibitors. This allows the release of NF-κB and its translocation to the nucleus where it associates with chromatin to activate the transcription of hundreds of genes. The best-studied NF-κB target genes include those encoding cytokines, chemokines, pro-survival factors, and IκBs as well (3).

Dysregulated NF-κB has been implicated in inflammatory disorders, autoimmune diseases and a variety of cancers (3,4). To prevent the detrimental consequences of excessive or prolonged NF-κB activation, many sophisticated negative regulatory mechanisms have been evolved to balance the strength and duration of NF-κB activity (4). It is well-known that activated NF-κB induces the re-synthesis of IκB, which can in turn relocate DNA-bound NF-κB back to the cytosol for the termination of NF-κB action (3,4). However, NF-κB-mediated transcription can be eventually terminated even in the absence of IκBs (5), indicating the significance of IκB-independent termination mechanisms. Among them, ubiquitination and proteasome-mediated degradation of DNA-bound NF-κB, particularly the RelA subunit, has been found to be a crucial complementary mechanism for timely termination of NF-κB-dependent transcription of inflammatory cytokines and chemokines (5–7). Recent findings that USP7-mediated deubiquitination of the RelA bound at the promoters of cytokine and chemokine genes stabilizes RelA and augments NF-κB-dependent transcription underscore the importance of ubiquitination of DNA-bound RelA in the termination of NF-κB activation (8,9). However, little is known about what triggers the ubiquitination and degradation of this form of RelA.

We have previously shown that upon stimulation with TNF-α or LPS, methyl transferase Set9, also called Set7, monomethylates RelA at K314/315 on the promoters of a subset of inflammatory cytokine genes, including IL-6 and IL-8, inducing proteasomal degradation of the DNA-bound RelA and promoting the termination of NF-κB-mediated transcription of these genes (10,11). However, the E3 ligase recognizing the methylated RelA remains uncharacterized. In this study, we identified that WSB1 and WSB2 (designated as WSB1/2 thereafter), two structurally similar WDR domain-containing members of the suppressor of cytokine signaling (SOCS) family that are known substrate-recognizing components of Cullin-RING E3 ligase (CRL) complexes (12,13), targeted K314/315-methylated RelA for ubiquitination and degradation. Our experimental and computational modeling data support a model that upon TNF-α stimulation, WSB1/2 are recruited to the chromatin and bind the chromatin-associated, lysine-methylated RelA in a WDR-dependent manner, thereby inducing K48-linked ubiquitination and degradation of RelA to facilitate timely termination of a subset of NF-κB target genes.

## Materials and methods

### Cell lines, plasmids and antibodies

U2OS, HepG2, HEK293T and NIH3T3 cells were obtained from American Type Culture Collection (ATCC) and tested negative for mycoplasma contamination. RelA-deficient NIH3T3 cells were reconstituted with wild-type (WT) RelA or RelA-K314/315R as described in our previous study (10). All cell types were cultured at 37°C under 5% CO_2_ in a humidified chamber in Dulbecco's modified Eagle's medium (DMEM) supplemented with 10% (v/v) fetal bovine serum except that U2OS cells were cultured in McCoy's 5a supplemented with 10% (v/v) fetal bovine serum.

Plasmids encoding T7-RelA, T7-RelA-K314/315R, T7-RelA-Y36A/E39D, Flag-Set9 and Flag-Set9-H297A were described previously (10). Plasmids encoding V5 tagged SOCS family of proteins were purchased from GE Dharmacon. PCR-based molecular cloning was performed to subclone Flag-WSB1, Flag-SOCS1/2 and Myc-WSB1/2 into a mammalian expression vector PCDNA3.1, GST-WSB1/2 and their mutant forms into a prokaryotic expression vector pGEX-4T-1 (GE Healthcare), and Flag-WSB1/2 into a viral vector pCDH-CMV-MCS-EF1-Puro-Vector (System Biosciences), respectively. The short hairpin RNAs of human WSB1 and WSB2 were inserted into pLKO.1-vector to construct shWSB1 and shWSB2 plasmids. The short hairpin RNA sequences are listed in Supplementary Table S1. Point mutations used in this study were generated by site-directed mutagenesis (Stratagene).

The antibodies used are as follows: rabbit anti-RelA (SC-372), mouse anti-RelA (SC-8008), mouse anti-Cul5 (sc-373822), rabbit anti-IκBα (SC-1643) and mouse anti-ubiquitin (SC-807) were from Santa Cruz; rabbit anti-K48-linked ubiquitin chain (8081S), rabbit anti-K63-linked ubiquitin chain (5621S), rabbit anti-RelA (8242S), rabbit anti-phospho-IκBα (2859S), rabbit anti-p38 (9212), rabbit anti-phospho-p38 (9215), rabbit anti-JNK (9252) and rabbit anti-phospho-JNK (4668), rabbit anti-GST (2622), rabbit anti-T7 (13246), rabbit anti-Biotin (HRP Conjugate) (7075S), rabbit anti-HA (3724S), rabbit anti-IgG (2729), and normal HRP-labeled secondary antibodies were from Cell Signaling Technology; rabbit anti-Flag (F7425), mouse anti-Flag (F1804), and Flag M2 agarose beads (A2220) were from Sigma; mouse anti-α-GAPDH (60004-1-Ig) and rabbit anti-H2A (10856-1-AP) were from Proteintech; Mouse anti-HA MagBeads (20566ES76), anti-T7 agarose beads (ab1230), and HRP-labeled secondary antibodies specific for the light chain (115-035-174) were from Yeasen Biotech, Abcam and Jackson, respectively. The anti-methylated RelA (Me-K314/315) antibody was used as previously described (11).

### Fractionation and TNF-α-induced ubiquitination of endogenous RelA

About 10^7^ cells were stimulated with TNF-α for 15 min. Cells were then washed and incubated in DMEM containing 20 μM MG-132 for 5 h before harvest. Cytoplasmic, nuclear soluble and chromatin-enriched fractions were prepared as previously described (14). Proteins in the chromatin-enriched fraction were extracted with extraction buffer (20 mM HEPES pH 7.9, 1 mM EDTA, 420 mM NaCl, 0.5% NP-40, protease inhibitor cocktail, 1 mM PMSF) by centrifuging at 4°C at 18 000 rcf for 20 min. Finally, protein extracts from 3 fractions were heated at 95°C for 5 min in the presence of 1% SDS, diluted 15–20 times with dilution buffer (20 mM HEPES pH 7.9, 1 mM EDTA, 150 mM NaCl, 0.5% NP-40, protease inhibitor cocktail, 1 mM PMSF), and concentrated with centrifugal filter devices (Amicon® Ultra-0.5ml, 10K) for RelA immunoprecipitation and immunoblotting with antibodies to ubiquitin, K48-, or K63-linked ubiquitin chains.

### Transfection and immunoblotting

All siRNAs used in this study (Supplementary Table S1) were synthesized in Shanghai GenePharma Co., Ltd and transfected with the Lipofectamine 3000 transfection reagent (Invitrogen, L3000015) according to the manufacturer's instructions. Plasmid transfection was conducted as described previously (10) or with PEI reagent (23966-2, Polysciences) according to the manufacturer's instructions. Immunoblotting was conducted as described previously (15) and quantitation of immunoblotting results were performed with Image J.

### Ubiquitination assay in transfected cells

HEK293T cells were transfected with plasmids encoding T7-His-RelA, HA-ubiquitin and other tagged Set9 and WSB1/2. After 30 h of transfection, cells were treated with proteasome inhibitor MG-132 (10 μM) for 5 h and lysed in denaturing lysis buffer (20 mM Tris–HCl pH 7.9, 1% SDS) at 95°C for 5 min. Heat-denatured cell lysates were first diluted 10–20 times with dilution buffer (20 mM Tris–HCl pH 7.9, 0.5 mM EDTA, 250 mM NaCl, 2 mM NEM, 0.5% NP-40, protease inhibitor cocktail, 1 mM PMSF) to reduce the concentration of SDS and then concentrated with centrifugal filter devices (Amicon® Ultra-0.5ml, 10K). Final lysates were subjected to either Ni-NTA sepharose (GE Healthcare) for affinity purification of His-tagged RelA or HA magnetic beads for immunoprecipitation of HA-tagged ubiquitin. To measure the level of RelA ubiquitination, Ni-NTA purified RelA was immunoblotted with anti-HA antibodies; HA immunoprecipitates were immunoblotted anti-T7 antibodies.

### GST pull-down assay

GST and various forms of GST-fused WSB1 or WSB2 were inducibly expressed in BL21 (DE3) *E. coli* cells and purified with Glutathione sepharose 4B beads (17-0756-01, GE Healthcare) as previously described (10). Whole cell lysates were prepared from HEK293T cells co-transfected with expression vectors for T7-RelA or T7-RelA-K314/315R alone or with Set9. For each pull-down assay, 1 μg beads-bound GST fusion protein and 50 μl HEK293T whole cell lysate were diluted in 500 μl lysis buffer, followed by rotation at 4°C for 1 h and immunoblotting with anti-T7 antibodies. For peptide competition assay, 100-fold molar excess of unmethylated RelA peptide (KSIMKKSPFS), the K314/315 monomethylated peptides (KSIMK[me1]K[me1]SPFS), or the K314/315 acetylated peptides (KSIMK[Ac]K[Ac]SPFS) was added before pull-down. To estimate the concentration of T7-RelA in whole cell lysates, T7 immunoprecipitates were prepared and separated in an SDS-PAGE gel together with a series of concentrations of BSA which has a molecular weight similar to T7-RelA. After staining of the gel, the concentration of T7-RelA in immunoprecipitates (Con^IP^) were estimated by band intensity-based comparison with BSA standard. The efficiency of immunoprecipitation (Eff^IP^) was estimated by comparing levels of T7-RelA on T7 immunoblot of the cell lysates and flow through from immunoprecipitation. The total concentration of T7-RelA in whole cell lysates (Con^Lys^) was calculated using the following equation: Con^Lys^= Con^IP^/Eff^IP^.

For Dot blot analysis of direct interactions of WDR-RelA peptides, unmodified and K314/315 monomethylated RelA peptides corresponding to amino acids 308–320 of RelA (TFKSIMKKSPFSG) were synthesized and N-terminally labeled with biotin in Kanglong Biotechnology Co., Ltd. To assess the binding of RelA peptides to WSB2, dot blot analysis was performed essentially as described elsewhere (16). Specifically, About 3 μg of glutathione beads-bound GST-WSB2 WDR protein was mixed with 1.8 nmol of each biotin-labeled RelA peptide in 500 μl GST binding buffer and incubated at 4°C for 1 h. Beads were washed with GST binding buffer for three times and 20 mM GSH was added to elute the GST-WSB2 WDR-peptide complex which was then denatured in the presence of 1% SDS and 2 mM DTT at 65°C for 5 min. Denatured samples were diluted and spotted onto a nitrocellulose membrane (P/N 66485, Pall Corporation) for dot blot analysis with anti-biotin and anti-GST antibodies.

### Viral transduction

Lentiviruses were packaged from HEK293T cells and used to infect U2OS and HepG2 cells. Infected cells were selected against puromycin to construct stable overexpression or knockdown cell lines. For double knockdown of WSB1 and WSB2 in U2OS cells, lentiviruses targeting WSB1 or WSB2 were combined at a 1:1 ratio before infection. Knockdown efficiency was measured by RT-PCR.

### RNA extraction, quantitative real-time PCR (RT-PCR) and RNA-sequencing

Total RNA was extracted with TRIzol (15596026, Invitrogen) following manufacturer's instructions. RT-PCR was conducted on a Roche system (LightCycler 480II) using SYBR Green reagent with the primers listed in Supplementary Table S1. All RT-PCR data were normalized against *GAPDH* and expressed as mean ± SD. Statistical differences between two groups were determined by unpaired two-tailed Student's t-test using Graphpad Prism (version 6.02). Nonlinear regression was chosen to compare the difference between gene expression patterns observed in control and WSB2 KD cells in the time course experiment. A p-value below 0.05 was considered statistically significant, and different levels of significance were expressed as follows: *P < 0.05; **P < 0.01; ***P < 0.001; ****P < 0.0001.

RNA-Sequencing was performed by Novogene, Beijing, China. Briefly, the RNA-seq library was prepared using Illumina kits, and the sequencing was performed using Illumina Novaseq 6000 by running 150 cycles. Differentially expressed genes were identified by ‘EdgeR’ R package; those with *P*-value < 0.05 and abs[log_2_ (fold change)]> 1 were considered significant. GSEA analysis was performed using R.

### Chromatin immunoprecipitation (ChIP)

ChIP assays were carried out as described previously (17). Briefly, U2OS cells were untreated or stimulated with TNF-α for 30 min and 60 min. After cross-linking, cells were sonicated until the chromatin was broken into fragments ranging from 500 to 800 bp in size. Immunoprecipitation was performed using antibodies against Flag, RelA, Me-RelA, Cul5 and equal amount of nonimmune IgG as control. The precipitated DNA fragments were examined by RT-PCR with primer sets listed in Supplementary Table S1. Results are expressed as the ratio of RT-PCR signal from ChIP with specific antibodies to that from mock ChIP with IgG.

### Immunofluorescence and confocal microscopy

U2OS cells stably expressing an empty vector, Flag-WSB1 or Flag-WSB2 were used for immunofluorescence which was performed as previously described (15) with a primary anti-Flag antibody (Sigma, F7425, at 1:200 dilution) and fluorescence-labeled secondary antibodies (Invitrogen, A21206, Alexa Fluor™ 488 Donkey Anti-Rabbit IgG, at 1:500 dilution). Microscopy of the antibody- and DNA probe DAPI-stained cells was performed with a Leica SP8 confocal microscope.

### Computational modeling

For apo WSB2 model, an unrefined structural model of human WSB2 was obtained from the AlphaFold2 database (18,19) using UniProt accession ID Q9NYS7. The model was placed into a 94 Å × 94 Å × 94 Å water box using the CHARMM-GUI webserver (20) with all the amino acids in their default protonation states, and the system neutralized with a final ion concentration of 0.15 M NaCl. Five replicas of the system were then equilibrated using NAMD2.14 (21) for 5 ns with protein backbone atoms restrained at 1 kcal/mol˙Å^2^, followed by a subsequent unrestrained equilibration of 5 ns. After equilibration each replica was simulated in NAMD3 (22) for 100 ns in order to obtain a consensus relaxed structure of WSB2.

For substrate-bound WSB2 model, the substrate RelA peptide bound to WSB2 was modeled after the bound peptide in a homologous template structure, methylated histone H3 tail bound to the WD repeat-containing protein 5 (WDR5, PDB: 2H9N) (23). To accurately place the substrate peptide, we first superimposed the equilibrated apo WSB2 onto the WDR5 crystal structure using the STAMP structural alignment tool in MultiSeq plugin (24,25) of Visual Molecular Dynamics (VMD) (26). The WSB2 substrate peptide was then modeled by adopting the backbone atoms of the superimposed histone H3 tail (sequence ARTK), while the side chains were modeled using the mutation function of VMD (26) to match the WSB2 recognized peptide sequence of RelA (312-IMKKS-316). In particular, the K314 residue of the WSB2 substrate peptide was modeled as a monomethylated lysine (MLZ residue in CHARMM protein topology (27,28) to reflect the experimentally characterized properties. The K314 atomic coordinates were directly adopted from those of the corresponding atoms in the arginine residue of the template structure, specifically, the N_ζ_ atom of K314 was mapped to the arginine's C_ζ_ so that the locations of their side chains’ charge centers remained identical, i.e. both in the proximity of a conserved aspartate at the binding site (D158 of WSB2 and D92 of WDR5). To keep the monomethylated lysine in the center of the modeled substrate peptide, an additional isoleucine residue was added to the N-terminus of the modeled backbone to form a bound pentapeptide, where both termini were modeled with neutral caps (acetylated N-terminal and N-methylamide at the C-terminal). Two replicates of the system were constructed from the modeled substrate-bound WSB2 and each was equilibrated for 6.25 ns unrestrained. Both replicates were then simulated each for 100 ns. Data analysis and molecular image rendering were done in VMD (26).

## Results

### Methylation of RelA by Set9 is critical for TNF-α-induced ubiquitination of chromatin-bound RelA

**Figure 1.**
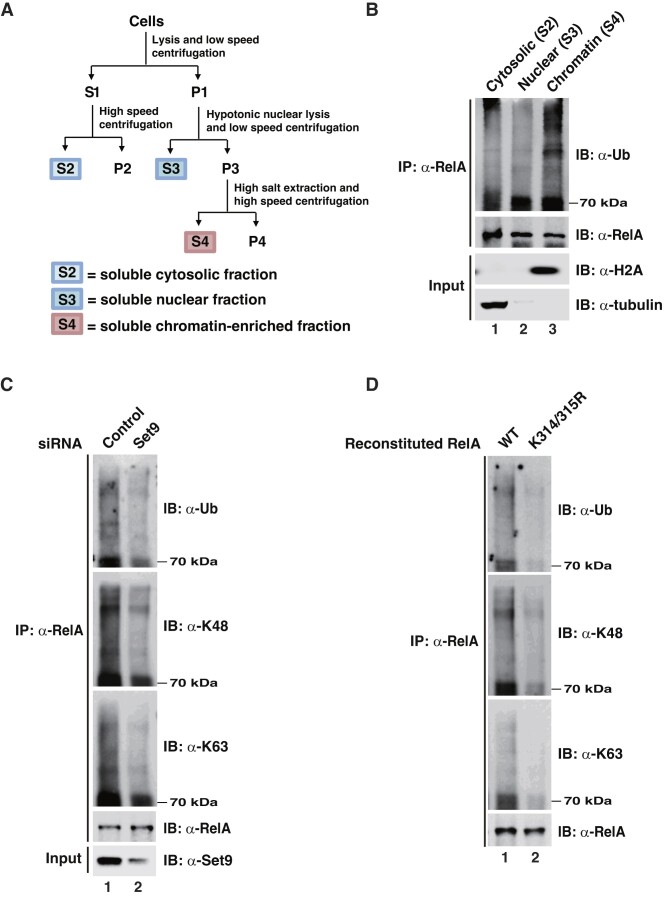
Methylation of RelA by Set9 is critical for ubiquitination of chromatin-bound RelA. (**A**) Schematic illustration of the fractionation process for the preparation of cytoplasmic (S2), nuclear (S3), and chromatin-enriched (S4) fractions. (**B**) NIH3T3 cells were pulse-stimulated with TNF-α for 15 min followed by treatment with proteasome inhibitor MG132 for 5 h. The cytoplasmic, nuclear, and chromatin-enriched fractions were prepared as in (A) and heat-denatured in the presence of 1% SDS for RelA immunoprecipitation (IP) and immunoblotting (IB) for ubiquitination. (**C**) NIH3T3 cells were transfected with control or Set9 siRNAs, and TNF-α-induced ubiquitination of RelA in chromatin-enriched fraction was assessed as described in (B) with antibodies against ubiquitin and K48-linked or K63-linked ubiquitin chains. (**D**) TNF-α-induced ubiquitination of RelA in chromatin-enriched fraction from RelA or RelA-K314/315R reconstituted RelA-deficient NIH3T3 cells was assessed as described in (C). The experiments in (B-C) were repeated 3 times, and those in (D) repeated twice. A representative result for each experiment is shown.

We have previously shown that Set9-mediated methylation of RelA at K314/315 triggered ubiquitination and degradation of DNA-bound RelA post NF-κB activation (10). To investigate whether endogenous RelA undergoes similar methylation-dependent ubiquitination and degradation after stimulation, we performed subcellular fractionation of TNF-α-treated NIH3T3 cells and evaluated the ubiquitination status of endogenous RelA immuneprecipitated from soluble cytosolic, nuclear and chromatin-enriched fractions (Figure 1A), largely representing the compartments containing inactivated, activated but DNA-unbound, and activated DNA-bound forms of NF-κB, respectively. We found that RelA from chromatin-enriched fraction was markedly ubiquitinated in response to TNF-α stimulation, while RelA from cytosolic and nuclear fractions was much less ubiquitinated (Figure 1B), indicating that during TNF-α stimulation RelA in the chromatin-enriched fraction is the major portion of RelA that undergoes ubiquitination. We also showed that LPS stimulation could induce ubiquitination of RelA in this fraction too (Supplementary Figure S1). To assess the effect of Set9-mediated methylation on TNF-α-stimulated RelA ubiquitination, we immuneprecipitated RelA from chromatin-enriched fraction isolated from TNF-α-stimulated Set9 knockdown cells to measure the levels of total ubiquitination and linkage-specific ubiquitination, including proteolytic K48-linked and non-proteolytic K63-linked ubiquitination. Interestingly, all three types of ubiquitination were markedly reduced in Set9 depleted cells compared with control cells (Figure 1C). Similarly, when we assessed the same types of RelA ubiquitination in RelA knockout cells reconstituted with WT or methylation-deficient mutant RelA-K314/315R, mutation of methylation sites resulted in decrease of all three types of ubiquitination on RelA (Figure 1D). These data suggest that Set9-mediated K314/315 methylation of chromatin-bound RelA might recruit an E3 ligase(s) for the ubiquitination, leading to either proteasomal degradation or non-proteolytic regulation of RelA.

### WSB1/2 promote the ubiquitination of methylated RelA

**Figure 2.**
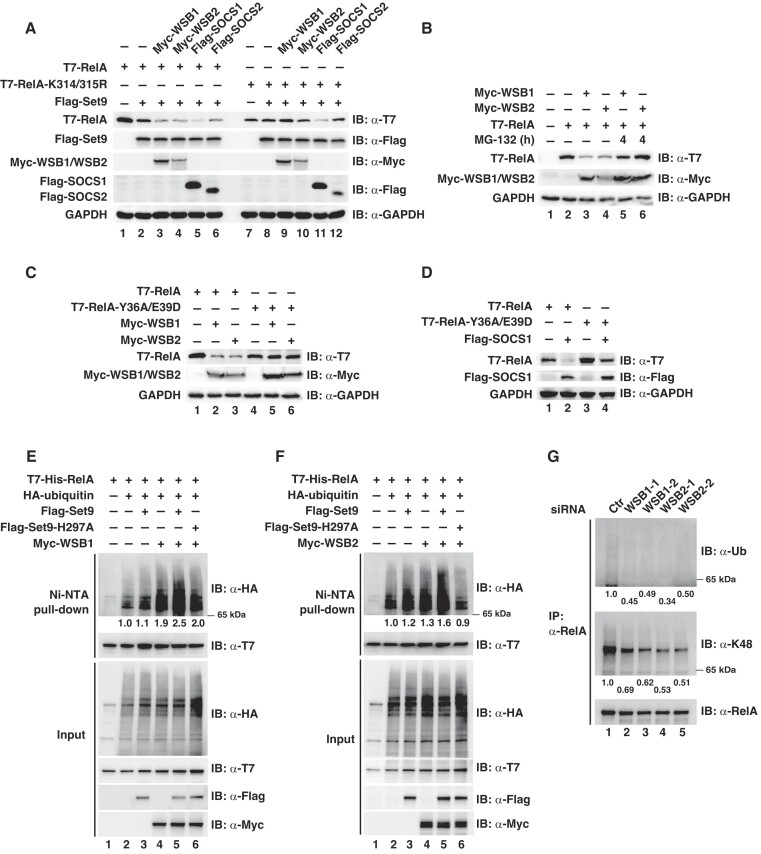
WSB1/2 promote the ubiquitination of methylated RelA. (**A**) HEK293T cells were transfected with plasmids encoding T7-tagged WT RelA or RelA-K314/315R and Flag-tagged Set9 together with 4 SOCS family proteins. Whole cell lysates were prepared and immunoblotted for indicated proteins. (**B**) HEK293T cells were transfected with the expression vectors for T7-RelA and Myc-tagged WSB1 or WSB2. At 24 h after transfection, cells were treated with MG-132 (20 μM) for 4 h, and whole cell lysates were immunoblotted with indicated antibodies. (**C, D**) HEK293T cells were transfected with plasmids encoding T7-tagged WT RelA or its DNA-binding defective mutant (RelA-Y36A/E39D) and Myc-tagged WSB1 or WSB2 **(C)**, or Flag-tagged SOCS1 (**D**); Whole cell lysates were prepared and immunoblotted with indicated antibodies. (**E, F**) HEK293T cells were transfected with plasmids encoding T7-His- RelA, HA-ubiquitin, Flag-tagged Set9 or catalytic inactive Set9-H297A together with Myc-tagged WSB1 (**E**) or WSB2 (**F**). After 30 h cells were treated with MG-132 for 5 h and heat-denatured in the presence of 1% SDS for affinity-purification with Ni-NTA resin. Purified RelA was immunoblotted with anti-HA antibodies for ubiquitination. Levels of ubiquitin, RelA, Set9 and WSB1/2 are shown as input in the lower panels. (**G**) U2OS cells were transfected with a control siRNA or 2 siRNAs against WSB1 or WSB2 and pulse stimulated with TNF-α for 15 min followed by treatment with MG-132 for 5 h. RelA was immunoprecipitated from denatured chromatin-enriched fraction and immunoblotted for total and K48-linked ubiquitination. Fold changes of RelA ubiquitination were normalized with the levels of immunoprecipitated RelA and shown below the blots (E–G). The experiments in (B–D) were repeated 3 times, and those in (A) and (E–G) repeated at least twice. A representative result for each experiment is shown.

To search for the E3 ligase(s) capable of ubiquitinating K314/315-methylated RelA, we designed a cell-based assay in which WT or methylation deficient mutant (K314/315R) of RelA were co-transfected with individual E3 ligases or components of E3 complexes into HEK293T cells, assuming that this specific E3 or it's essential component(s) only promotes the ubiquitination and degradation of WT RelA but not RelA-K314/315R mutant. Using this assay, we performed a screen in the SOCS family of proteins that are best-known as substrate receptors in CRL E3 complexes (12,13) and are well-known negative regulators of immune receptor- or cytokine receptor-mediated signaling (29). One member of this family, SOCS1, has been identified as a CRL substrate receptor targeting RelA for degradation (30–32). Expectedly, co-transfection of SOCS1 reduced the protein levels of RelA and RelA-K314/315R, suggesting that SOCS1-mediated ubiquitination and degradation of RelA is likely independent on K314/315 methylation (Figure 2A). Interestingly, two closely related SOCS family members, WSB1 and WSB2, which have highly conserved amino acid sequence and domain structures (12), reduced the levels of WT RelA but not RelA-K314/315R mutant (Figure 2A). In contrast, other members of the SOCS family were either unable to reduce the levels of RelA or capable of reducing the levels of both WT RelA and RelA-K314/315R (Supplementary Figure S2). These data suggest that methylation on K314/315 might account for the reduction of RelA levels by WSB1/2. This WSB1/2-mediated reduction of RelA was due to proteasome-mediated protein degradation since WSB1/2 failed to reduce the level of RelA in the presence of proteasome inhibitor MG-132 (Figure 2B). These results suggest that WSB1/2 are the potential CRL substrate receptors targeting K314/315-methylated RelA for its ubiquitination and degradation.

We have previously shown that methylation-dependent degradation of RelA occurs on chromatin-bound RelA (10,11), raising a possibility that WSB1/2 might specifically induce the degradation of chromatin-bound RelA. Supporting this, we found that DNA-binding deficient RelA mutant (RelA-Y36A/E39D) was resistant to WSB1/2-mediated degradation (Figure 2C). In contrast, SOCS1 was still able to degrade RelA regardless of the DNA-binding status of RelA (Figure 2D).

Next, we investigated if WSB1/2 induced the ubiquitination of RelA and whether Set9-mediated methylation of K314/315 promoted such ubiquitination. To this end, transfected cells were heat-denatured and His-tagged RelA was pulled down with Ni-NTA resin followed by immunoblotting for ubiquitin. We found that co-expression of RelA with Set9 or WSB1/2 alone slightly or moderately enhanced ubiquitination of RelA, but the level of ubiquitinated RelA was further increased when RelA was co-expressed with both Set9 and WSB1 or WSB2 (Figure 2E and F). However, co-expression of the enzymatically inactive form of Set9 (H297A) with WSB1 or WSB2 failed to increase the ubiquitination of RelA (Figure 2E and F). Expectedly, when opposite pulldowns of HA-tagged ubiquitin from the same lysates were performed to assess RelA ubiquitination, we observed similar results (Supplementary Figure S3), further supporting the importance of K314/315 methylation for WSB1/2-mediated RelA ubiquitination. To evaluate the effect of WSB1/2 on the ubiquitination of endogenous RelA, we used siRNAs to knock down the expression of WSB1 or WSB2, respectively, in U2OS cells (Supplementary Figure S4) and examined TNF-α-induced ubiquitination of RelA in chromatin-enriched fraction as in Figure 1. The ubiquitination of RelA, particularly the K48-linked ubiquitination, was slightly reduced in WSB1 knockdown cells but moderately reduced in WSB2 knockdown cells (Figure 2G). This result indicates that both WSB1 and WSB2 are involved in the ubiquitination and degradation of chromatin-bound endogenous RelA with WSB2 being dominant over WSB1 under this condition. The difference in the ubiquitinated RelA from WSB1 or WSB2 knockdown cells likely reflected the different expression levels of WSB1 and WSB2, with the levels of WSB2 3–4 times higher than WSB1 in U2OS and HepG2 cells used in this study (Supplementary Figure S5).

### WSB1/2 interact with methylated K314/315 of RelA through their WDR domains

**Figure 3.**
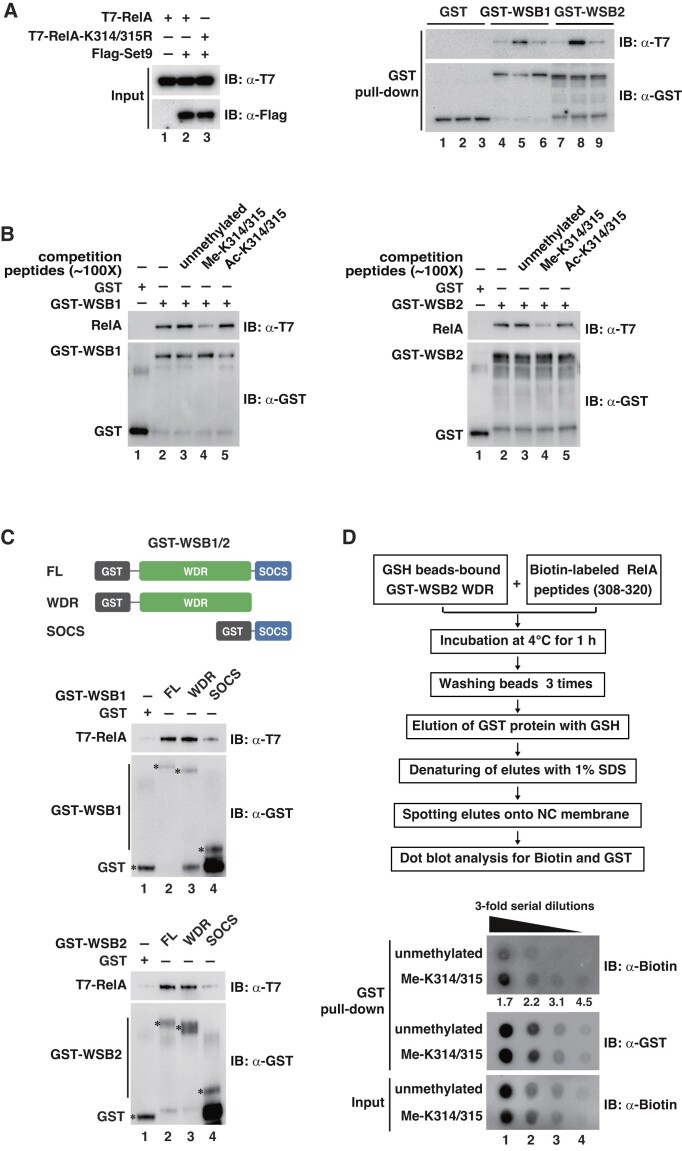
WSB1/2 interact with methylated K314/315 of RelA through their WDR domains. (**A**) GST pull-down assay was performed with recombinant GST, GST-WSB1 or GST-WSB2 and whole cell lysates from HEK293T cells transfected with T7-tagged WT RelA or RelA-K314/315R and Flag-tagged Set9 as indicated. The associated RelA was assessed by IB with anti-T7 antibodies. (**B**) Approximately 100-fold excess molar of unmethylated or K314/315-methylated RelA peptides (a.a. 308–320) was added in GST pull-down assay performed with whole cell lysate from HEK293T cells transfected with T7-tagged WT RelA and Flag-tagged Set9. RelA associated GST-WSB1 (left) or GST-WSB2 (right) was assessed as in (A). (**C**) GST pull-down assay with full-length (FL), WDR or SOCS domains of WSB1 or WSB2 and the whole cell lysate from HEK293T cells transfected with T7-tagged WT RelA and Flag-tagged Set9 was performed as in (A). Asterisks to the left of each band on the two GST blots indicate the proteins of GST and GST-fusions of WSB1 or WSB2. (**D**) GSH sepharose beads-bound GST-WSB2 WDR protein was incubated with biotin-labeled unmethylated or K314/315-methylated RelA peptides (a.a. 308–320) and the GST-WSB2 WDR protein was eluted with GSH and the associated peptides were spotted onto a nitrocellulose membrane and assessed via dot blot analysis with anti-biotin and anti GST antibodies. Ratios of methylated peptides to unmethylated peptides bound to GST-WSB2 WDR at each dilution were quantitated and shown below the blot. The experiments in (A), (C) and (D) were repeated at least 3 times, and those in (B) repeated twice. A representative result for each experiment is shown.

To define the molecular mechanism by which WSB1/2 promote the ubiquitination of RelA in a methylation-dependent manner, GST pull-down assays were performed to explore if WSB1/2 bind to methylated RelA. GST-tagged recombinant WSB1 or WSB2 was incubated with cell lysates prepared from HEK293T cells co-transfected with Set9 and WT RelA or RelA-K314/315R and the pull-down products were immunoblotted for associated RelA. Both GST-tagged WSB1 and WSB2, but not GST alone, pulled down a high level of WT RelA from lysates co-transfected with Set9 (Figure 3A). However, neither WSB1 nor WSB2 pulled down the RelA-K314/315R mutant which cannot be methylated by Set9 (Figure 3A). When a synthetic RelA peptide of 13 amino acids containing unmethylated, methylated, or acetylated K314/315 residuals were utilized to compete with methylated full-length RelA in the GST pull-down assays, WSB1 and WSB2 pulled down much less RelA with the addition of the methylated peptides, but not with the unmethylated or acetylated peptides (Figure 3B). These data suggest that binding of WSB1 or WSB2 to RelA specifically depends on methylation of K314/315.

Both WSB1 and WSB2 are composed of an N-terminal WDR domain and a C-terminal SOCS domain (Figure 3C). The WDR of WDR5 has been shown to be critical for specific recognition of methylated lysine on histone H3 (23,33), raising a possibility that the WDRs of WSB1/2 might be involved in the binding to methylated K314/315. Supporting this hypothesis, we found that full-length or the WDR-containing region but not the SOCS domains of WSB1 and WSB2 bound to the methylated RelA in the GST pull-down assay (Figure 3C). To further confirm this methylation-dependent interaction, direct binding of the WDR domain to K314/315-methylated RelA was tested with an *in vitro* dot blot assay (Figure 3D). When synthetic biotin-labeled unmethylated or K314/315-methylated RelA peptides were incubated with the same amount of glutathione beads-bound GST-WSB2 WDR protein and the associated RelA peptides were eluted and spotted on a nitrocellulose membrane for dot blot assay, we observed that the WDR domain of WSB2 bound more K314/315-methylated peptides compared to the unmethylated peptides (Figure 3D). This *in vitro* binding assay further demonstrates that the WDR of WSB2 directly associates with RelA with an approximately 3–4-fold higher affinity for K314/315 methylated form than the unmethylated form.

### WSB2 negatively regulates the expression of NF-κB target genes

**Figure 4.**
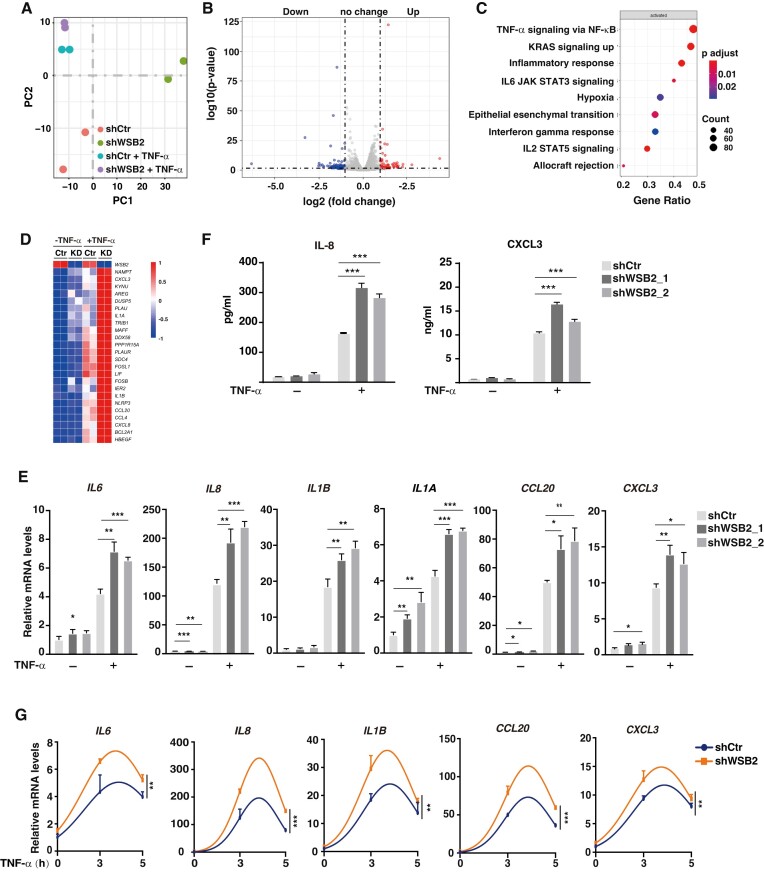
WSB2 negatively regulates the expression of NF-κB target genes. (**A–D**) U2OS cells transduced with a control shRNA or a WSB2 shRNA were stimulated with or without TNF-α for 5 h and total RNA was extracted for bulk RNA-sequencing. (**A**) Principal component analysis of the sequencing results. (**B**) Scatter plotting of up-regulated [log_2_ (fold change) >1], down-regulated [log_2_ (fold change) < 1], and unchanged [ 1 < log2 (fold change) <1] genes in control and WSB2-KD U2OS cells 5 h after TNF-α stimulation. (**C**) Gene set enrichment analysis was performed to determine the most significantly up-regulated pathways in TNF-α-stimulated WSB2-KD cells. (**D**) A heat map showing *WSB2* and the top 24 NF-κB target genes up-regulated in TNF-α-stimulated WSB2-KD cells. (**E**) U2OS cells transduced with control or two different WSB2 shRNAs were stimulated with TNF-α for 5 h and the expression of indicated genes was measured by quantitative RT-PCR. (**F**) Levels of TNF-α-induced IL-8 and CXCL3 proteins were measured by ELISA in U2OS cells transduced with control or WSB2 shRNAs. (**G**) U2OS cells transduced with control or WSB2 shRNAs were stimulated with TNF-α for indicated time points and the expression of the indicated genes was measured by quantitative RT-PCR. Results in (E), (F) and (G) are shown as means ± SD from triplicate experiments. *P < 0.05; **P< 0.01; ***P < 0.001. Statistical analysis was performed using unpaired two-tailed Student's *t*-test for (E) and (F), and nonlinear regression for (G).

While WSB1 and WSB2 bound to K314/315 methylated RelA and induced its ubiquitination and degradation to a similar extent when over-expressed in cells or assessed in the GST-pulldown assay (Figures 2A, C, E, F, 3A, C), endogenous WSB2 appeared to be dominant over WSB1 in terms of its ability to ubiquitinate RelA and the expression level in cultured cells, including U2OS and HepG2 cells (Figure 2G, Supplementary Figure S5). Therefore, we focused on WSB2 for the remaining of our study. To determine the potential role of WSB2-mediated ubiquitination of RelA in modulating NF-κB-dependent transcription, we performed RNA sequencing of WT and WSB2-knockdown (KD) U2OS cells (Supplementary Figure S6) with or without TNF-α stimulation. Principal components analysis of the sequencing result revealed distinct transcriptional profile in WT cells compared to KD cells in the presence or absence of TNF-α (Figure 4A). Statistical analysis indicated that upon TNF-α stimulation, WSB2 knockdown resulted in the up-regulation of 98 genes and the down-regulation of 130 genes (abs[log_2_ (fold change)] > 1 and *P*-value < 0.05) (Figure 4B). Gene set enrichment analysis of differentially expressed genes revealed that no pathway was down-regulated to a significant level (*P*-adjust > 0.05) (Supplementary Table S2), but 9 pathways were significantly augmented in WSB2-KD cells, among which the NF-κB signaling pathway was the most significant one with the largest number of up-regulated genes (Figure 4C). As shown in the heat map, dozens of TNF-α-induced NF-κB target genes were elevated in WSB2-KD cells (Figure 4D), supporting a negative regulatory role of WSB2 on NF-κB target gene expression.

To further verify the transcriptomic data from the RNA-seq, we quantified the expression of some of the up-regulated NF-κB target genes, including *IL6, IL8, IL1A, IL1B, CCL20* and *CXCL3*, by real-time PCR. Consistent with the RNA-seq result, knockdown of WSB2 using two different shRNAs elevated TNF-α-induced expression of these genes (Figure 4E). A similar increase in protein levels was also observed for IL-8 and CXCL3 in TNF-α-stimulated WSB2-KD cells (Figure 4F). Moreover, a time course experiment demonstrated that WSB2 knockdown resulted in not only elevated but also sustained expression of NF-κB target genes (Figure 4G). Nevertheless, regulation by WSB2 seems to be gene-specific since TNF-α-induced transcription of other NF-κB target genes, e.g. *TNFRSF9*, was not significantly affected by WSB2 knockdown (Supplementary Figure S7). Similar up-regulation of these NF-κB target gene expression was also observed in WSB2-depleted HepG2 cells (Supplementary Figure S8), indicating that negative regulation of NF-κB by WSB2 might not be a cell-type specific phenotype. Similar to TNF-α stimulation, LPS-stimulated expression of NF-κB target genes was also increased in WSB2 knockdown cells (Supplementary Figure S9), which is consistent with our previous discovery that both TNF-α and LPS can induce Set9-dependent RelA methylation for the termination of NF-κB activation (10,11). Collectively, these data indicate that WSB2 negatively regulates the transcription of a subset of NF-κB target genes, likely through Set9-mediated methylation of K314/315 of RelA.

### WSB2 is recruited to the promoters of NF-κb target genes

**Figure 5.**
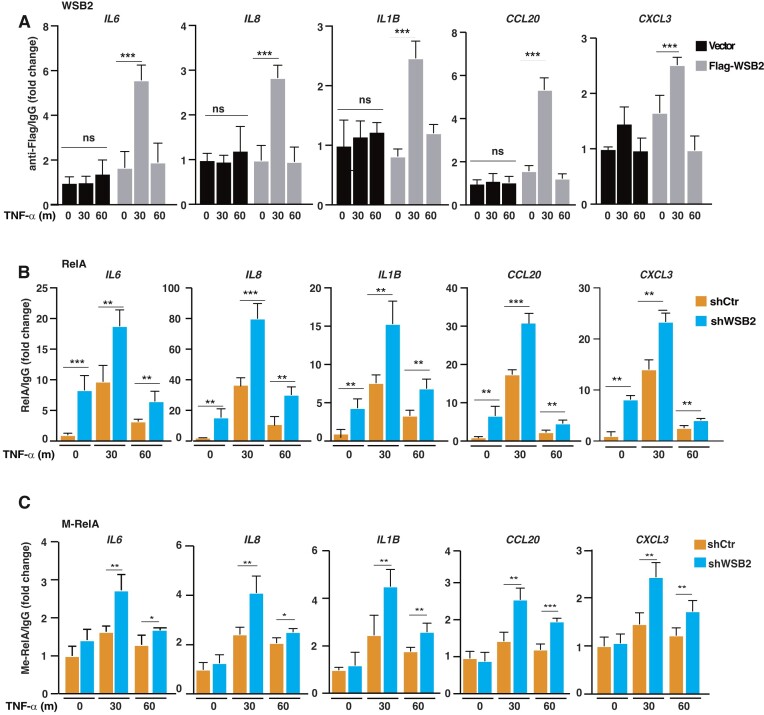
WSB2 is recruited to the promoters of NF-κB target genes. (**A**) U2OS cells stably expressing vector or Flag-tagged WSB2 were stimulated with TNF-α for indicated time points. ChIP assays were performed using anti-Flag antibody or non-specific mouse IgG antibodies and probed for the promoters of indicated genes. (**B, C**) U2OS cells stably expressing control or WSB2 shRNAs were stimulated with TNF-α for indicated time points. ChIP assays were performed with antibodies against RelA (**B**) or methylated K314/315 of RelA (**C**) and probed for the promoters of indicated genes. Results are expressed as the ratio of RT-PCR signal from ChIP with specific antibodies to that from mock ChIP with IgG and shown as means ± SD from triplicate experiments. * *P* < 0.05; ** *P* < 0.01; *** *P* < 0.001; ns, no significant. Statistical analysis was performed using unpaired two-tailed Student's *t*-test.

To investigate the regulation of chromatin-bound RelA by WSB2, we next employed ChIPs to assess the recruitment of WSB2 to the promoters of NF-κB target genes. Due to the lack of ChIP-quality WSB2 antibodies, we generated U2OS cell lines stably expressing Flag-tagged WSB2 or an empty vector, which mimicked the constitutive expression of WSB2 in U2OS cells (Supplementary Figure S12). ChIPs with anti-Flag antibodies revealed that WSB2 was recruited to the promoters of WSB2-regulated NF-κB target genes, including *IL6, IL8, IL1B, CCL20* and *CXCL3*, 30 min after TNF-α stimulation (Figure 5A), a time point when strong K314/315 methylation signal could be detected (10,11). Importantly, when we assessed the recruitment of endogenous Cul5, the scaffold protein that WSB1/2 interact with to form a CRL E3 complex (34), to the promoters of NF-κB target genes, we found that TNF-α also stimulated the association of Cul5 with the promoters of WSB2-regulated genes, such as *CXCL13* and *CCL20* (Supplementary Figure S13), providing indirect evidence that endogenous WSB2 might be recruited to the promoters of NF-κB target genes upon stimulation.

When determining if WSB2 regulates chromatin-bound RelA, we performed ChIP assays and observed that WSB2 knockdown significantly enhanced the levels of both total RelA and K314/315-methylated RelA at the promoters of WSB2-regulated genes (Figure 5B, C), but did not seem to alter the levels of total nuclear RelA under the same condition (Supplementary Figure S14), supporting a major role for WSB2 in controlling chromatin-bound RelA. For *TNFRSF9*, whose expression was not regulated by WSB2 (Supplementary Figure S7), we detected no recruitment of WSB2 to its promoter in Flag-WSB2 expressing cells upon TNF-α stimulation (Supplementary Figure S15). We didn’t detect accumulation of methylated RelA at its promoter in WSB2-KD cells either (Supplementary Figure S16). Together, these data suggest that TNF-α stimulates WSB2 association with the promoters of WSB2-regulated genes where it targets chromatin-bound methylated RelA for degradation.

### Computational modeling reveals the preferential binding of WSB2 to methylated RelA

**Figure 6.**
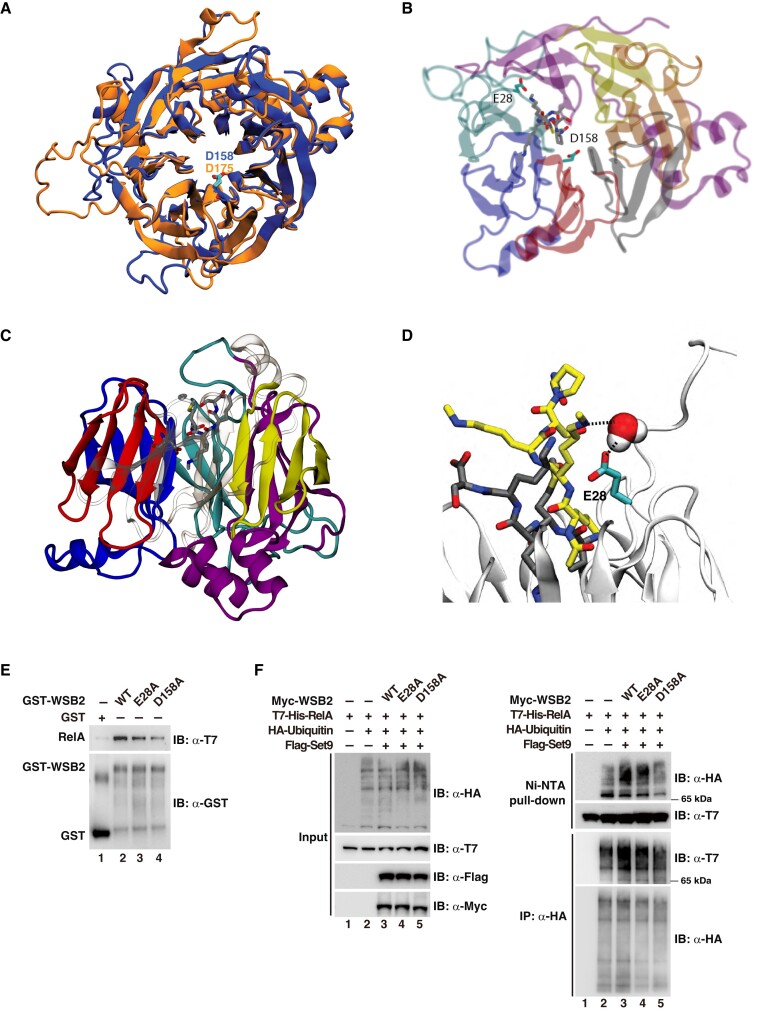
Computational modeling and experimental verification of the interaction between WSB2 and methylated RelA. (**A**) Alignment of WSB1 (orange) and WSB2 (blue) structures predicted by AlphaFold2. D158 in the WDR domain of WSB2 and D175 in the WDR domain of WSB2 are perfectly overlapped. (**B**) Structural model of human WSB2 in complex with a K314 monomethylated RelA peptide (312-IMKme1KS-316). D158 in Repeat 3 of WSB2 WDR domain coordinates methylated K314. (**C**) A side view of the model shown in (B). (**D**) Mechanistic sketch generated by Marvin Sketch showing that the Nϵ atom of monomethylated K314/315 forms a hydrogen bond with a water molecule which in turn forms a hydrogen bond with the carboxylate oxygen of E28 in WSB2. (**E**) GST pull-down assay with GST, GST-tagged WT WSB2, WSB2-E28A or WSB2-D158A and the whole cell lysate from HEK293T cells transfected with T7-tagged RelA and Flag-tagged Set9 was performed as in Figure 3C. (**F**) HEK293T cells were transfected with indicated plasmids and treated with 10 μM MG-132 for 5h before harvesting. After lysis under a denaturing condition, one half of each lysate was used for Ni-NTA purification and ubiquitination assay as in Figure 2E, and the other half was immunoprecipitated with anti-HA antibodies for immunoblotting with anti-T7 antibodies. Levels of tagged ubiquitin, RelA, Set9 and WSB2 are shown as input in the lower panels. The experiments in (E) were repeated 3 times, and those in (F) repeated twice. A representative result for each experiment is shown.

To gain more detailed mechanistic insight into the recognition of methylated K314/315 by the WDR domain of WSB1/2, we performed modeling of the binding of WSB1/2 to synthetic RelA peptides monomethylated at single lysines (K314 or K315) or at both lysines (K314/K315), based on the well-established interaction between histone H3K4me2 and the WDR domain of WDR5 (23). Since experimental structures of WSB1/2 are not available, their predicted structures were obtained from the AlphaFold2 database (18). The overall structures of WSB1 and WSB2 exhibited a high level of similarity when aligned (Figure 6A). WDRs of WSB1/2 were dominated by a seven-bladed β-propeller structure with each blade composed of a four-stranded anti-parallel beta-sheet (Figure 6A). These WDRs are similar to the typical WDR domains of other WD-40 proteins, including WDR5 (23).

A modeled K314-monometylated RelA peptide (312-IMKme1KS-316) was docked onto the WSB2 structure. We found that the aspartate residue at position 158 (D158) in Repeat 3 of the WDR domain of WSB2, which is conserved between WSB1 (D175) and WDR5 (D92) coordinated a basic residue (K314 or K315) on the monomethylated RelA peptide (Figure 6B & C). When a peptide with both K314 and K315 mono-methylated (312-IMKme1Kme1S-316) was used in the simulation, methylated K314/315 were able to interact with D158, but disassociated rapidly, indicating an unstable binding between D158 and methylated K314/315 (data not shown). We also noticed an interaction between the side chain of K314 with E28 in WSB2 via a bridging water molecule in the monometylated context (Figure 6D). The nitrogen atom of monomethylated K314 or K315 side chain forms a hydrogen bond with a water molecule, which in turn forms a hydrogen bond with the carboxylate oxygen of E28 in WSB2 (Supplementary Figure S17). This water-mediated hydrogen bond network seems to be similar to the water molecular-bridged interaction between H3K4me2 and E322 in WDR5 (23).

To further confirm the findings from the computational modeling and simulation, we replaced E28 or D158 of WSB2 with an alanine and assessed the abilities of these mutants to bind and ubiquitinate RelA. Comparing to WT WSB2, both E28A and D158A mutants displayed reduced methylation-dependent WSB2-RelA interaction in the GST pulldown assay with D158A causing a stronger reduction of the interaction (Figure 6E). Consistent with the defects in their bindings to methylated RelA, D158A mutant displayed remarkably reduced ability to induce the ubiquitination of RelA while E28A mutant partially affected the ubiquitination of RelA by WSB2 (Figure 6F). Together, the computational modeling and experimental data provide a molecular basis for how methylation of K314 or K315 enhances WSB1/2-RelA interaction and support the notion that WSB1/2 preferentially targets methylated RelA for ubiquitination and degradation.

## Discussion

Ubiquitination and proteasomal degradation of DNA-bound RelA has been demonstrated to be a critical mechanism for the termination of activated NF-κB (5–9). We have previously shown that Set9-mediated methylation of K314/315 triggered this process through an uncharacterized E3 ligase (10). In the present study, we identified WSB1 and WSB2 as critical regulators that recognize the methylated lysines via their corresponding WDR domains and ubiquitinate methylated RelA on the promoters of a subset of NF-κB target genes, thereby attenuating NF-κB-mediated transcription. These findings establish a sequential modification-dictated cascade consisting of lysine methylation, ubiquitination and degradation that are executed by Set9 (writer), WSB1/2 (readers) and the proteasome (effector), respectively, favoring our previously proposed NF-κB code hypothesis for the regulation of NF-κB target gene expression, an analogy to histone code hypothesis for gene expression (10).

In general, ubiquitination of a protein requires a prior modification that can be either phosphorylation or hydroxylation (35). We and another group have first reported that methylation could be a third modification that primes proteins, including RelA and DNMT1, for ubiquitination (10,36,37). Since then, an increasing number of studies have shown that lysine methylation catalyzed by Set9 or other methyltransferases induces ubiquitination and degradation of various transcription factors or chromatin-associated adaptor proteins, including SOX2, E2F1, SMARCC1, SMARCC2, FOXO1, FOXO3, SMAD7, UHRF1 and β-catenin (38–46). These findings indicate that ubiquitination-dependent protein degradation is a common outcome of lysine methylation. In consistent, lysine demethylation catalyzed by demethylases, like LSD1, stabilizes proteins (40,45,47,48).

How lysine methylation on a protein is read and interpreted by other proteins has been intensively studied on histones (49,50). Reader proteins with various methylation-binding domains, including Chromo, MBT, Tudor, PHD and WDR, have been identified. They recognize methylated lysines on histones in a site- and state-specific manner to modulate chromatin structure and gene expression (16,51,52). How methylated lysine on non-histone proteins is recognized by E3 ubiquitin ligases to trigger ubiquitination has been elusive. Recent studies demonstrated that MBT domain-containing protein L3MBTL3 destabilized methylated proteins by functioning as a substrate-recognizing subunit of CRL4^DCAF5^. The MBT domain of L3MBTL3 is able to specifically bind to monomethylated lysine on DNMT1 (53), SOX2 (39) and SMARCC1/2 (41) for the recruitment of CRL4^DCAF5^, which in turn ubiquitinates these proteins for degradation. Interestingly, the same methylated lysine residuals on DNMT1 and SOX2 could be recognized by Tudor domain of PHF20L1, which competes with L3MBTL3 for the binding to DNMT1 and SOX2, thereby protecting against L3MBTL3-CRL4^DCAF5^-mediated destabilization (48,53), suggesting a dynamic and complex regulation of the consequence of lysine methylation. Our study identifies WDR domain-containing WSB1/2 as novel lysine methylation-recognizing components of CRL E3 complexes that preferentially target a methylated protein for ubiquitination and proteasomal degradation.

Analogous to L3MBPL3, WSB1 has been shown to be a substrate recognizing subunit of CLR E3 complex with the SCOS box domain associating with Cul5 and the WDR domain binding to the substrate (34). WSB1 is able to target HIPK2 (54), VHL (55), RhoGDI2 (56) and ATM (57) for ubiquitination and degradation in caner development and progression and build K27- and K29-linked ubiquitin chains on LRRK2 for protein aggregation in the pathogenesis of Parkinson's disease (58), suggesting a broad substrate specificity of WSB1. By contrast, little is known about WSB2 as a substrate receptor in any E3 complex. It is quite possible that WSB2 might also associate with the catalytic core complex Elongin B/C-Cul5-Rbx1, serving as a substrate recognizing subunit of the large E3 complex, to ubiquitinate their substrates including methylated RelA.

Our computational modeling results revealed a conserved mode in which the WDR domain binds to K/R-containing peptide (R2 in histone H3 and K314 or K315 in RelA) through a conserved aspartate residual and distinguishes methylation status of the same (RelA) or neighboring (K4 in histone H3) lysine by providing a hospitable electrostatic environment for the methylated species. Our simulations suggest that RelA binding to WSB2 is mediated via a conserved E28 which interacts with the peptide via a bridging water molecule as in the case of the homologous WDR5. Unexpectedly, the WDR domains of WSB1/2 prefer single methylated K314 or K315 than methylation of both lysines. Our previous *in vitro* methylation assay demonstrated that K314 and K315 could be singly or doubly methylated (10,11). Given the nature of Set9-mediated methylation reaction, it is predictable that K314 and K315 are separately methylated and some singly methylated RelA molecules can be further methylated on the other site to allow double methylation on K314/315. Accordingly, we reason that within cells, WSB2 or WSB1 binds to RelA once a single lysine (K314 or K315) gets methylated.

It's well known that ubiquitination, either proteolytic or non-proteolytic, plays profound roles in activating and terminating the NF-κB signaling pathway (59,60). A growing body of evidence reveals that NF-κB subunits themselves are also subject to ubiquitination which serves as an important mechanism to control transcription of NF-κB target genes (7,61). In the case of the RelA subunit, multiple E3 ligases have been identified to induce its ubiquitination and degradation in the cytoplasm or in different compartments within the nucleus before and/or after NF-κB activation. PPARγ (62), ING4 (63) and RNF182 (64) have strong E3 activities toward RelA for K48-linked ubiquitination before and after NF-κB activation, implying that they induce RelA degradation without distinguishing between inactive and active forms of RelA. In contrast, a RING-like domain-containing protein PDLIM2 and a RING domain E3 MKRN2 can function alone or in synergy in the nucleus to ubiquitinate RelA for its translocation from the nucleoplasm to the nucleolus for proteasomal degradation (65,66). Interestingly, the cullin-RING ubiquitin ligase (CRL) complex ECS^SOCS1^, in which the substrate receptor SOCS1 cooperates with accessory proteins COMMD1 and GCN5 to associate with Cul2 and Elongins B/C, targets chromatin-bound RelA for ubiquitination and degradation after NF-κB activation (30–32,67,68). Our study identified WSB1/2 as a new type of RelA-targeting E3 components that mainly target chromatin-bound RelA, acting in a manner distinct from ECS^SOCS1^: (i) ECS^SOCS1^ prefers to target Ser468-phosphorylated RelA (67,69), but ECS^WSB1/2^ preferentially target K314 or K315 methylated RelA; (ii) SOCS1 requires accessory factors COMMD1 and GCN5 to strongly associate with RelA (31,67,69), WSB1/2 appear to be able to directly bind to methylated RelA by themselves and (iii) ECS^SOCS1^ and ECS^WSB1/2^ regulate different sets of NF-κB target genes with the former mainly inhibiting non-cytokine genes such as *ICAM1*, *Mx1* and *CD86* (67,68) and the latter suppressing cytokine genes including *IL1A*, *IL1B*, *IL6*, *IL8*, *CCL20* and *CXCL3*. Therefore, these two E3 complexes may play non-redundant roles in terminating NF-κB-dependent transcription by targeting chromatin-bound RelA in gene- and RelA modification-specific manner.

Our *in vitro* experiments showed that WSB1 and WSB2 had a remarkable similarity in protein structure and ability to bind and ubiquitinate methylated RelA. Compared to the effect of WSB2 knockdown on gene expression, fewer NF-κB target genes such as *IL8 and IL1A* were up-regulated in WSB1 knockdown cells; other WSB2-regulated genes, for instance *IL6* and *IL1B*, remained unaltered upon WSB1 knockdown (Supplementary Figure S10). This is likely due to the differential expression levels of WSB1 and WSB2 in U2OS and HepG2 cells, where WSB1 had a lower level of expression than WSB2 (Supplementary Figure S5) and therefore less effect on RelA ubiquitination than WSB2 (Figure 2G). However, for the genes down-regulated by both WSB1 and WSB2, *IL1A* and *IL8*, double knockdown of WSB1 and WSB2 further enhanced their expression levels, compared with each single knockdown of them (Supplementary Figure S11). These findings imply that WSB1 and WSB2 may function redundantly in regulating the transcription of some NF-κB target genes, although WSB2 appeared to have a dominant role over WSB1 in the cell types we studied.

A recent study showed evidence for the cytoplasmic localization of WSB1 in Bel-7402 cells stably expressing a Flag-tagged WSB1 (70). When we evaluated the subcellular localization of WSB2 and WSB1 as well by immunofluorescence in U2OS cells that stably expressed Flag-tagged WSB2 or WSB1, we observed that WSB2 and WSB1 were distributed widely in the cell with a higher level of WSB1 in the nucleus and a higher level of WSB2 in the cytoplasm regardless of the stimulation with TNF-α (Supplementary Figure S18). While expressed at a lower level in the nucleus, it might be sufficient for WSB2 to regulate chromatin-bound RelA and gene expression mediated by NF-κB. In contrast, WSB2 and WSB1 did not seem to get involved in TNF-α-induced upstream signaling events occurring in the cytoplasm, as depleting either of them had little effect on phosphorylation and degradation of IκBα, activation of MAP kinases p38 and JNK (Supplementary Figure S19), and the nuclear translocation of RelA (Supplementary Figure S14).

Our ChIP experiments indicated that stably expressed WSB2 was recruited to promoters of WSB2-regulted NF-κB target genes in response to TNF-α stimulation, resembling the TNF-α-induced recruitment of Cul5 (Supplementary Figure S13). However, whether the recruitment of WSB2 and Cul5 to chromatin for RelA ubiquitination is dependent on RelA methylation or other mechanisms awaits further studies.

In summary, the data presented in this study define a unique NF-κB terminating mechanism by which a pair of WDR domain-containing E3 substrate receptors, WSB1/2, preferentially recognize and ubiquitinate chromatin-bound lysine methylated RelA for proteasomal degradation. This study offers new insight into gene-specific regulation of NF-κB-dependent transcription and provides novel potential targets for treatment of aberrant NF-κB activation-elicited inflammation.

## Supplementary Material

gkae161_Supplemental_Files

## Data Availability

The RNA-seq data have been deposited into the NCBI Gene Expression Omnibus with an accession number GSE233286. The computational modeling data have been deposited to ModelArchive (modelarchive.org) with an accession codes ma-1h7fl. All other data are available upon reasonable request.
