# Supplementary material for: WSB1/2 target chromatin-bound lysine-methylated RelA for proteasomal degradation and NF-κB termination
Source: Nucleic Acids Res. 2024 Mar 7;52(9):4969–84. doi: 10.1093/nar/gkae161 (PMC11109945; doi:10.1093/nar/gkae161)
Supplement: gkae161_Supplemental_Files [file gkae161_supplemental_files.zip › Original scans of Western blots.pdf]

Fig 1

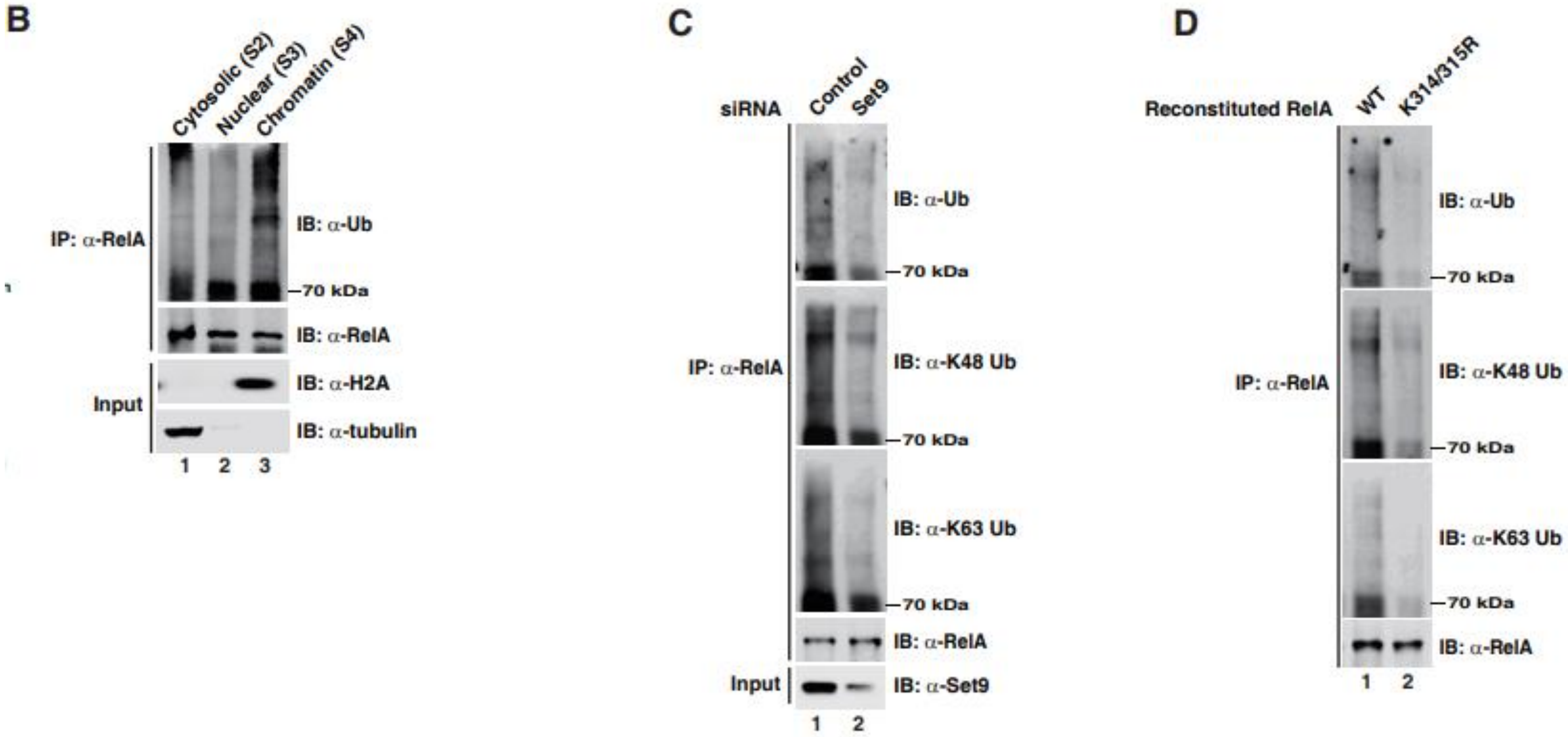
B
Cytosolic (S2)
Nuclear (S3)
Chromatin (S4)
IP: α-RelA
IB: α-Ub
70 kDa
IB: α-RelA
Input
IB: α-H2A
IB: α-tubulin
1 2 3
C
siRNA
Control
Set9
IP: α-RelA
IB: α-Ub
70 kDa
IB: α-K48 Ub
70 kDa
IB: α-K63 Ub
70 kDa
IB: α-RelA
Input
IB: α-Set9
1 2
D
Reconstituted RelA
WT
K314/315R
IP: α-RelA
IB: α-Ub
70 kDa
IB: α-K48 Ub
70 kDa
IB: α-K63 Ub
70 kDa
IB: α-RelA
1 2

Fig 1B

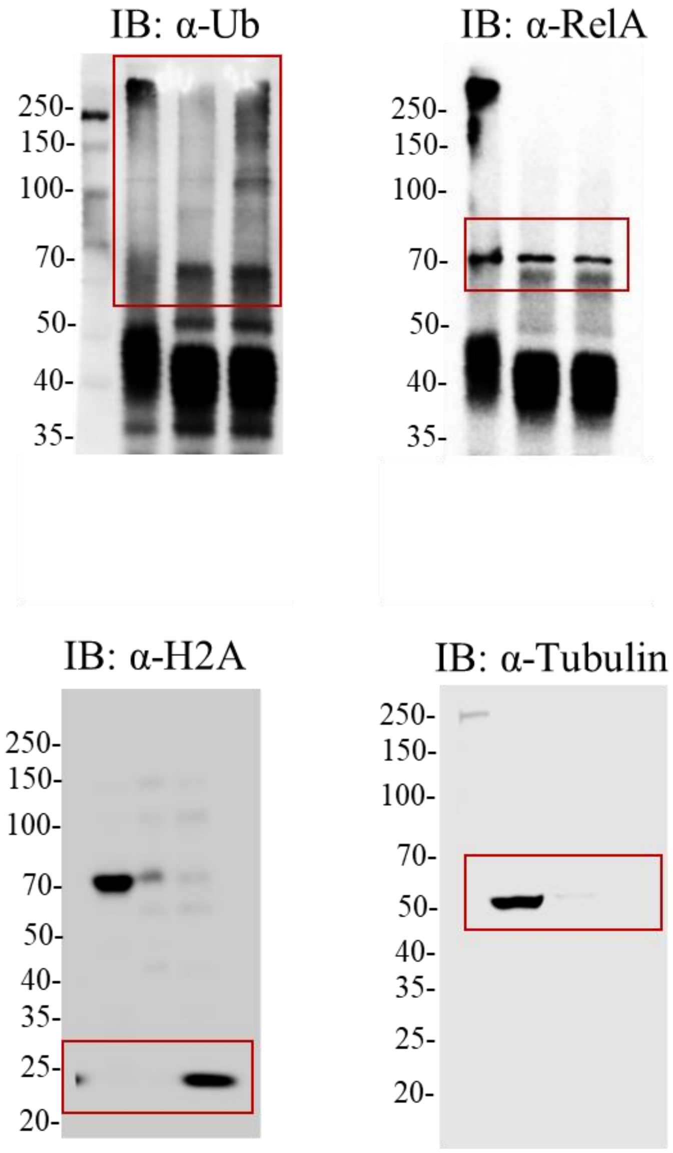

Fig 1C

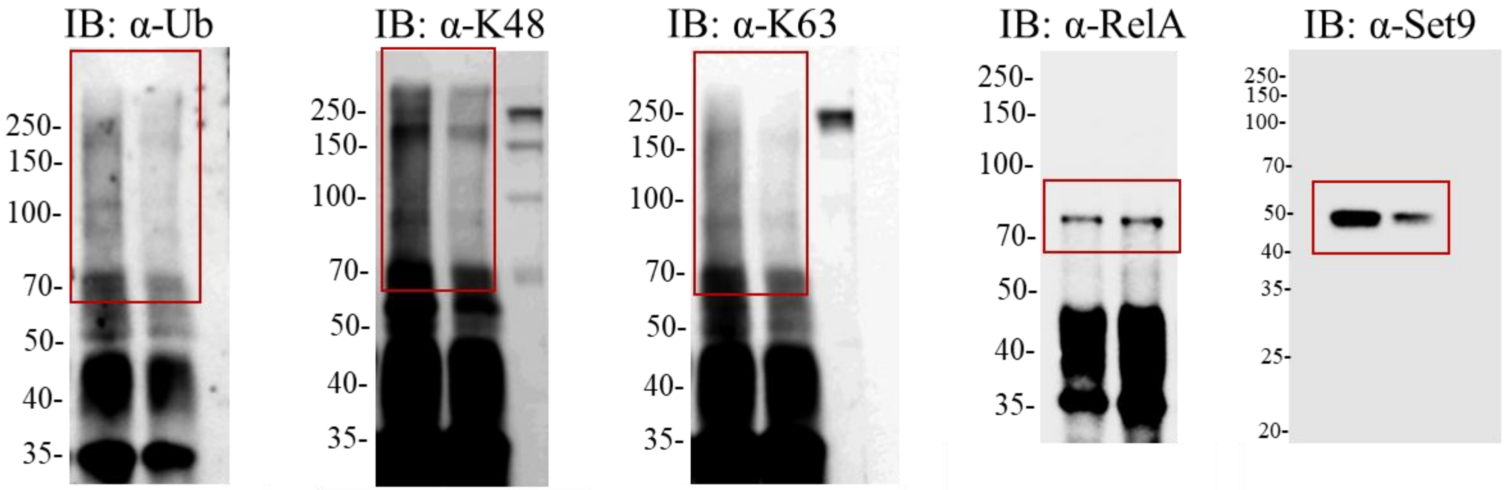

Fig 1D

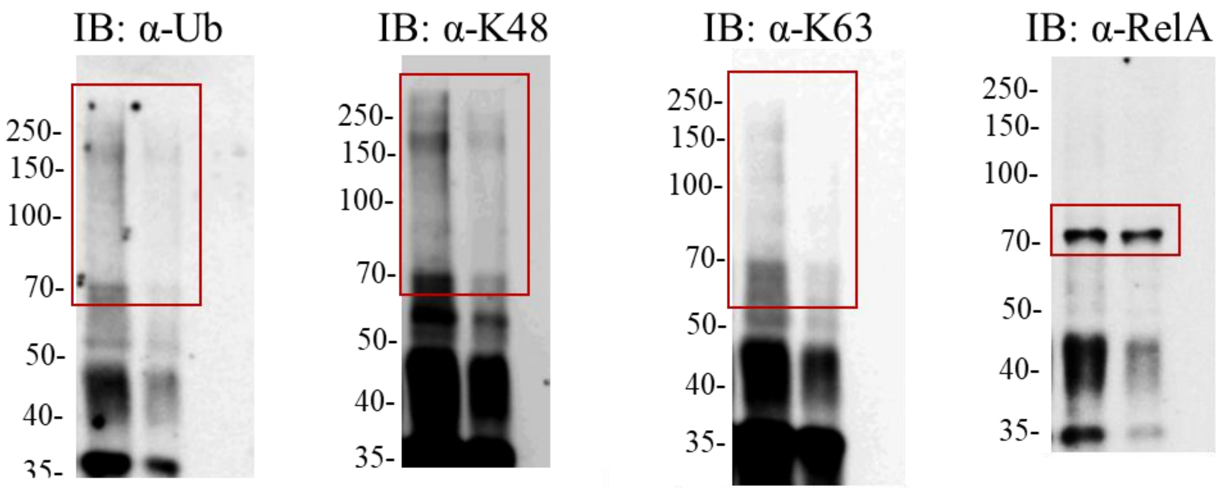

Fig 2A

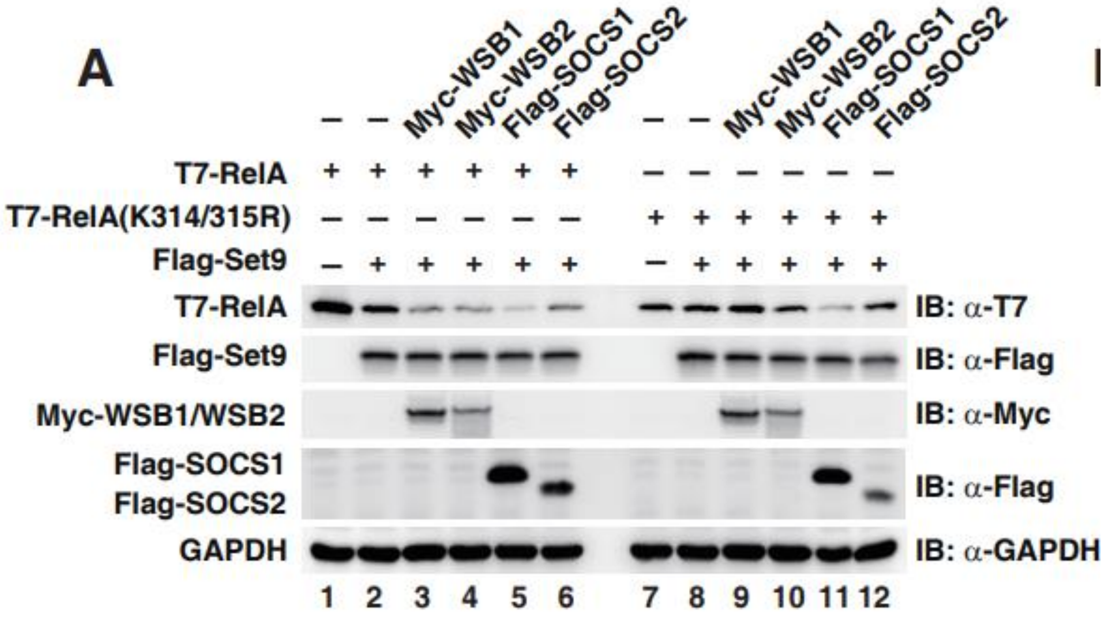
A
Myc-WSB1
Myc-WSB2
Flag-SOCS1
Flag-SOCS2
T7-RelA
T7-RelA(K314/315R)
Flag-Set9
T7-RelA
Flag-Set9
Myc-WSB1/WSB2
Flag-SOCS1
Flag-SOCS2
GAPDH
IB: α-T7
IB: α-Flag
IB: α-Myc
IB: α-Flag
IB: α-GAPDH
1 2 3 4 5 6 7 8 9 10 11 12

Fig 2A

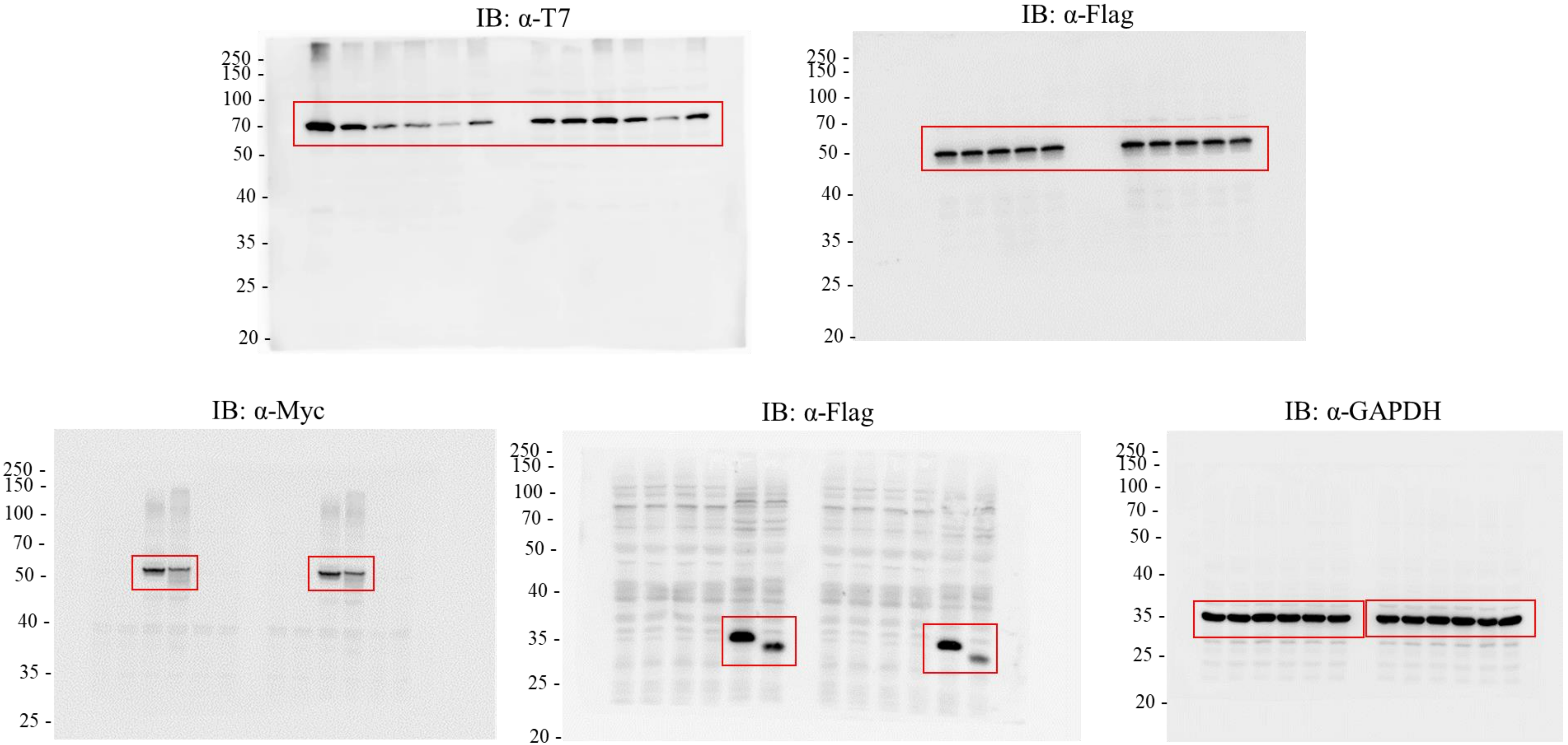
IB: α-T7
250 -
150 -
100 -
70 -
50 -
40 -
35 -
25 -
20 -
IB: α-Flag
250 -
150 -
100 -
70 -
50 -
40 -
35 -
25 -
20 -
IB: α-Myc
250 -
150 -
100 -
70 -
50 -
40 -
35 -
25 -
IB: α-Flag
250 -
150 -
100 -
70 -
50 -
40 -
35 -
25 -
20 -
IB: α-GAPDH
250 -
150 -
100 -
70 -
50 -
40 -
35 -
25 -
20 -

Fig 2 B-D

B

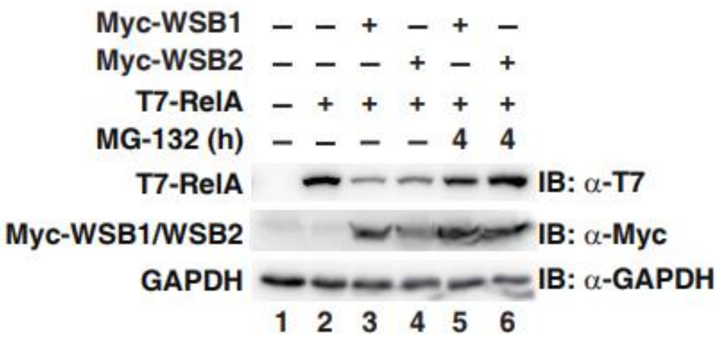

Myc-WSB1 – – + – + –
Myc-WSB2 – – – + – +
T7-RelA – + + + + +
MG-132 (h) – – – – 4 4
T7-RelA
IB: α-T7
Myc-WSB1/WSB2
IB: α-Myc
GAPDH
IB: α-GAPDH
1 2 3 4 5 6

C

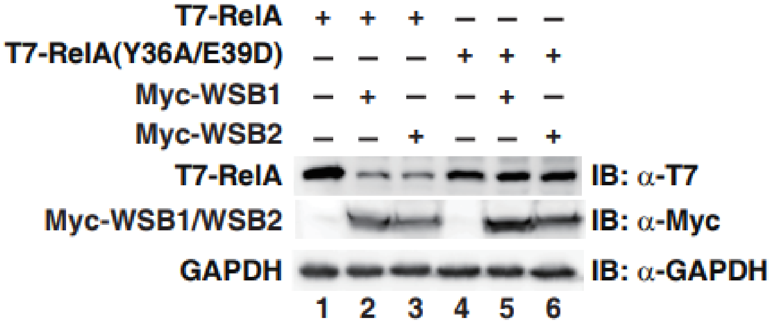

T7-RelA + + + – – –
T7-RelA(Y36A/E39D) – – – + + +
Myc-WSB1 – + – – + –
Myc-WSB2 – – + – – +
T7-RelA
IB: α-T7
Myc-WSB1/WSB2
IB: α-Myc
GAPDH
IB: α-GAPDH
1 2 3 4 5 6

D

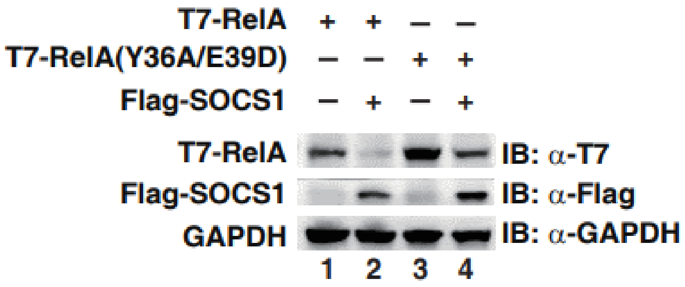

T7-RelA + + – –
T7-RelA(Y36A/E39D) – – + +
Flag-SOCS1 – + – +
T7-RelA
IB: α-T7
Flag-SOCS1
IB: α-Flag
GAPDH
IB: α-GAPDH
1 2 3 4

Fig 2B

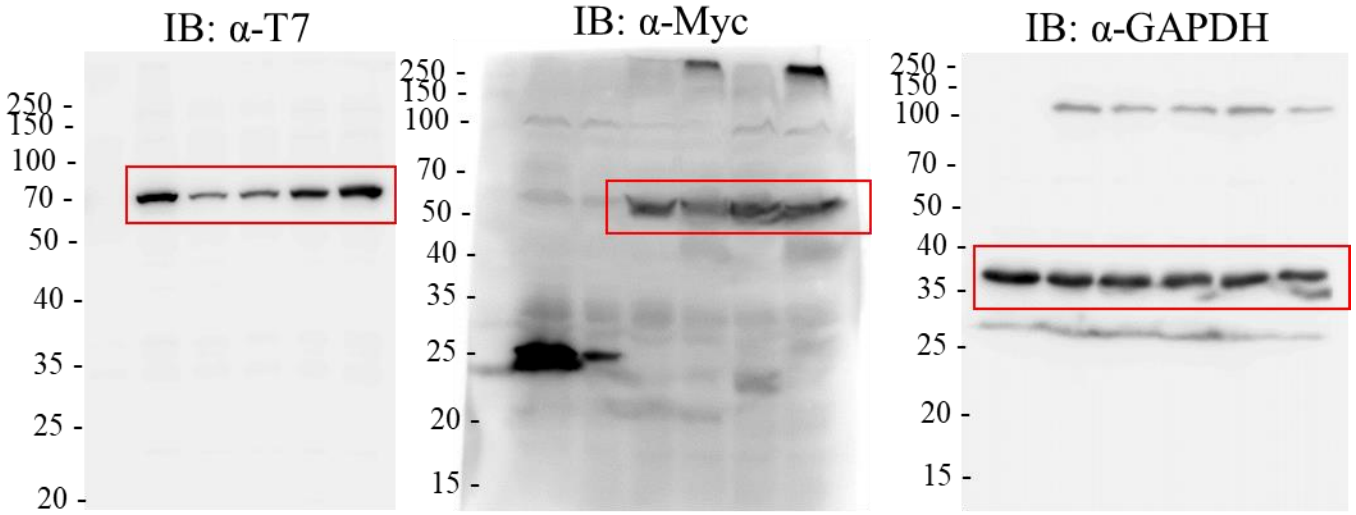

Fig 2D

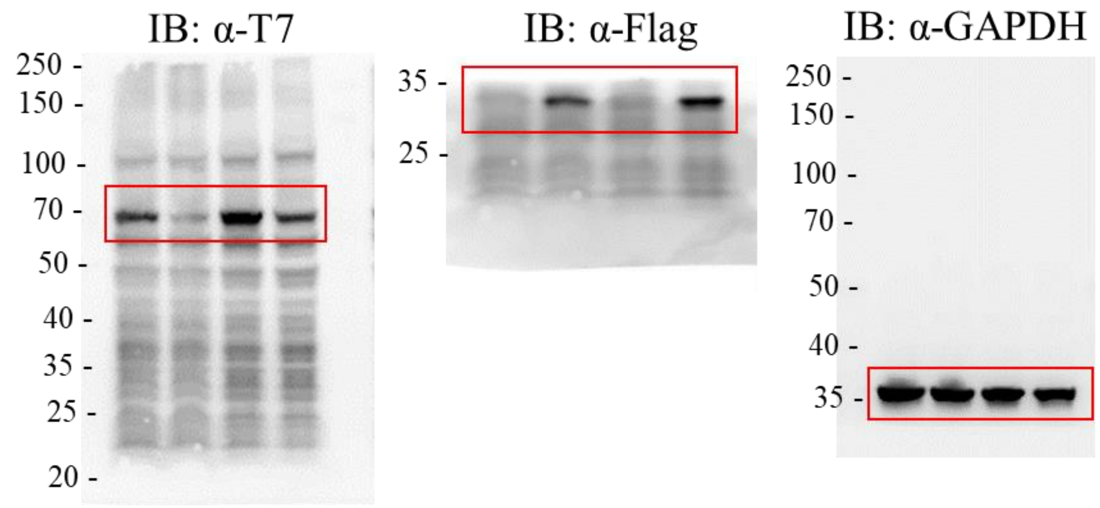

Fig 2C

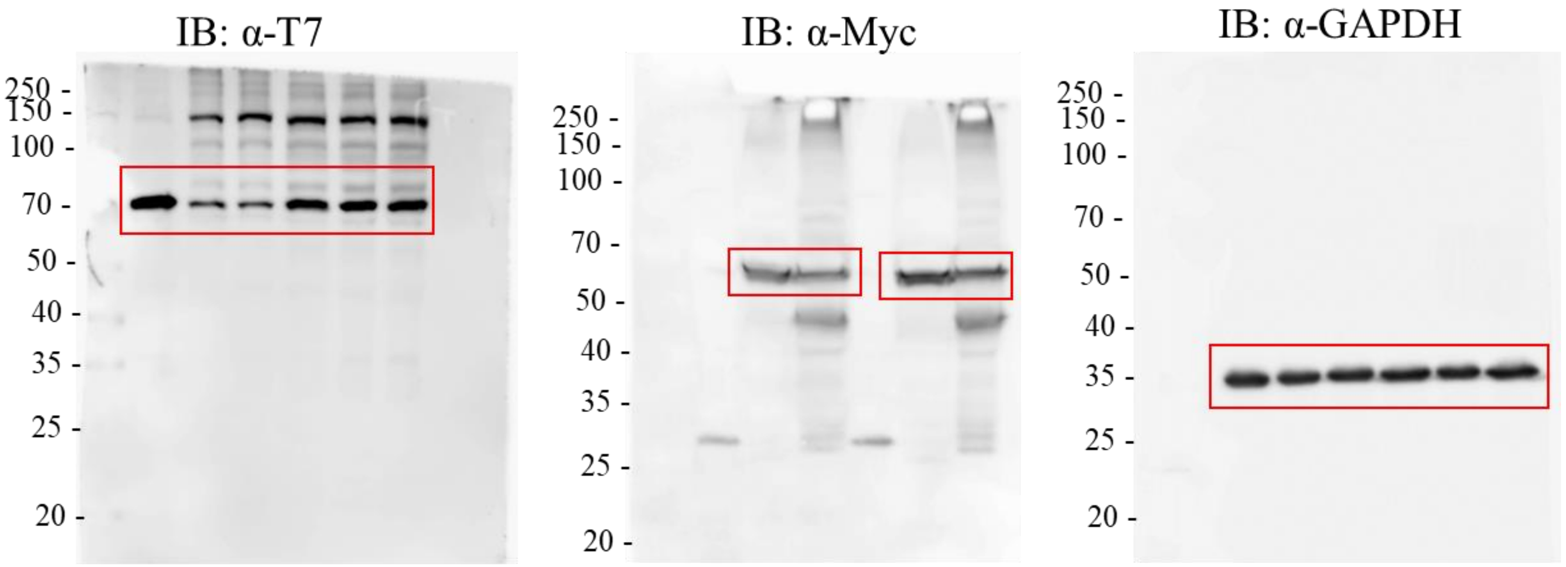

Fig2 E

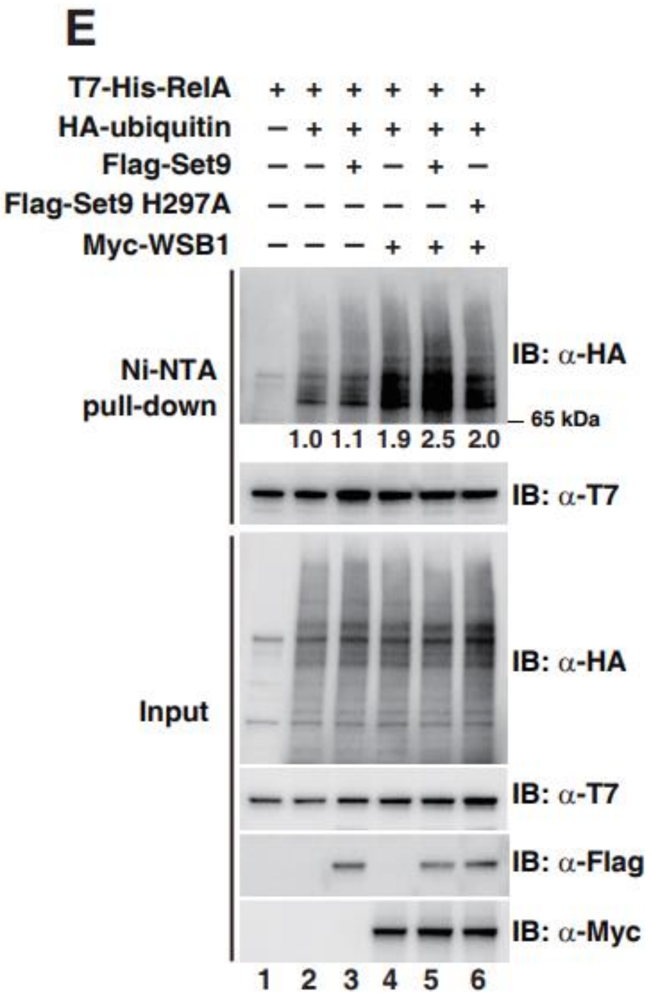

E
T7-His-RelA + + + + + +
HA-ubiquitin − + + + + +
Flag-Set9 − − + − + −
Flag-Set9 H297A − − − − − +
Myc-WSB1 − − − + + +
Ni-NTA pull-down
IB: α-HA
65 kDa
1.0 1.1 1.9 2.5 2.0
IB: α-T7
Input
IB: α-HA
IB: α-T7
IB: α-Flag
IB: α-Myc
1 2 3 4 5 6

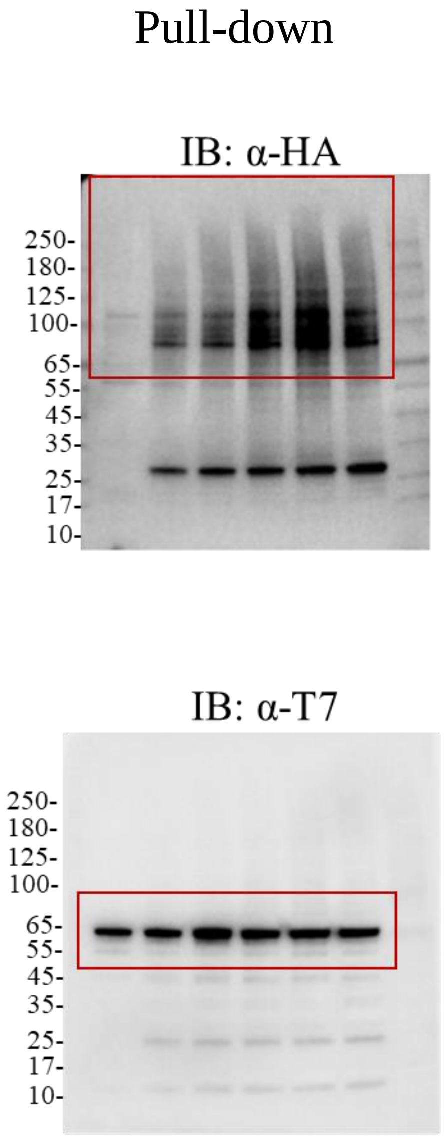

Pull-down
IB: α-HA
250- 180- 125- 100- 65- 55- 45- 35- 25- 17- 10-
IB: α-T7
250- 180- 125- 100- 65- 55- 45- 35- 25- 17- 10-

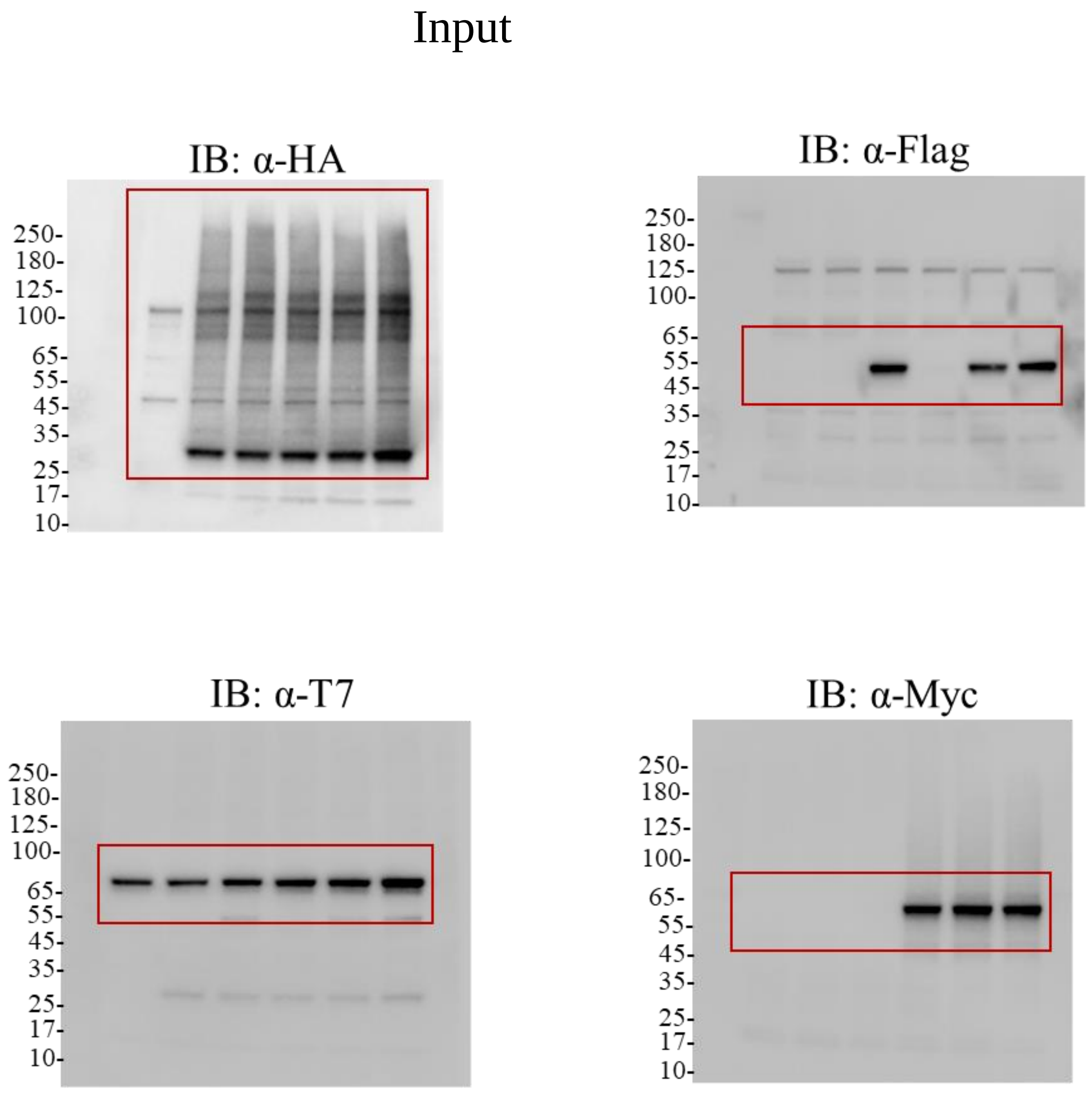

Input
IB: α-HA
250- 180- 125- 100- 65- 55- 45- 35- 25- 17- 10-
IB: α-Flag
250- 180- 125- 100- 65- 55- 45- 35- 25- 17- 10-
IB: α-T7
250- 180- 125- 100- 65- 55- 45- 35- 25- 17- 10-
IB: α-Myc
250- 180- 125- 100- 65- 55- 45- 35- 25- 17- 10-

Fig2 F

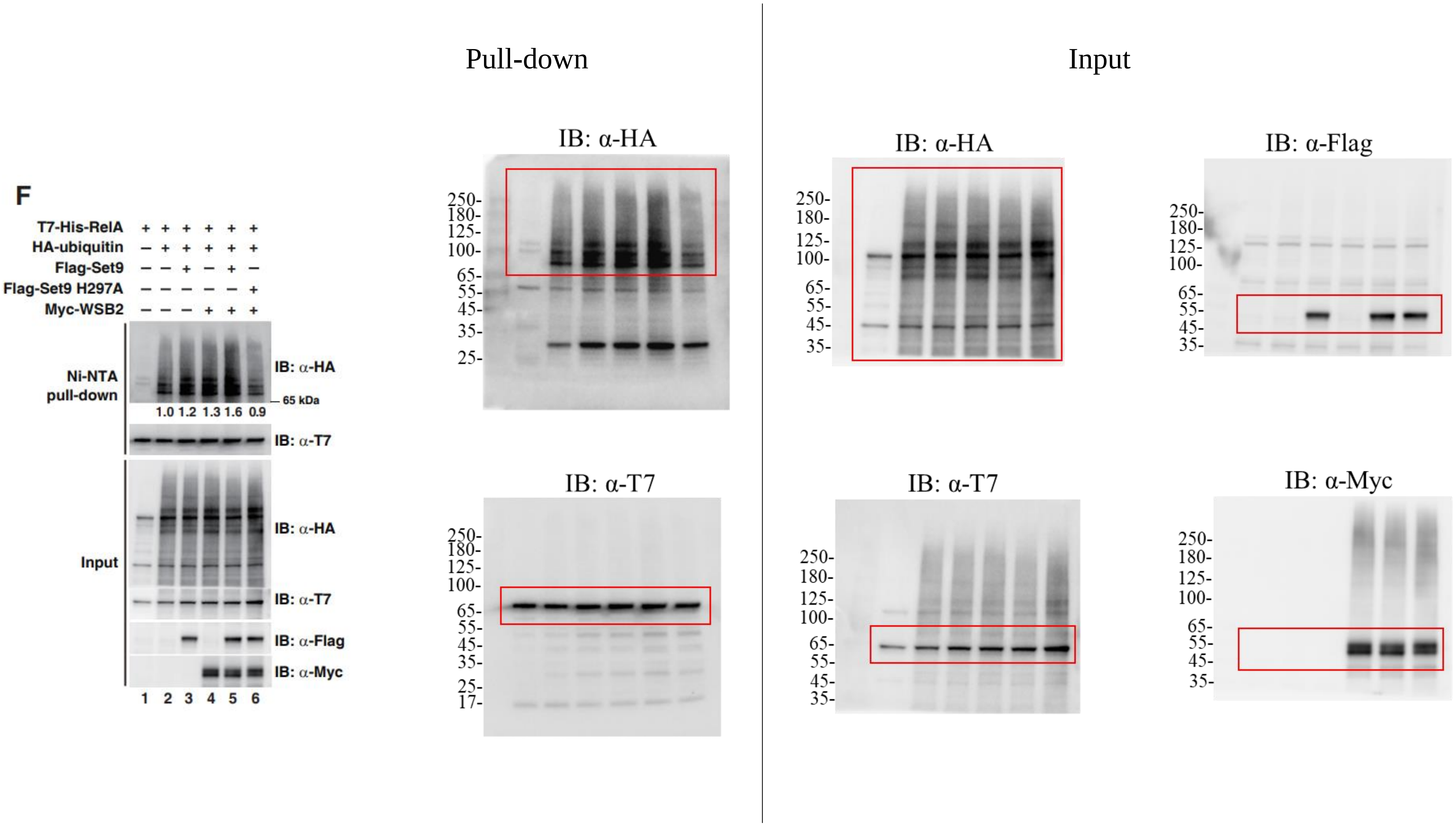

Pull-down
Input
F
T7-His-RelA + + + + + +
HA-ubiquitin − + + + + +
Flag-Set9 − − + − + −
Flag-Set9 H297A − − − − − +
Myc-WSB2 − − − + + +
Ni-NTA pull-down
IB: α-HA
65 kDa
1.0 1.2 1.3 1.6 0.9
IB: α-T7
Input
IB: α-HA
IB: α-T7
IB: α-Flag
IB: α-Myc
1 2 3 4 5 6
IB: α-HA
250-
180-
125-
100-
65-
55-
45-
35-
25-
IB: α-T7
250-
180-
125-
100-
65-
55-
45-
35-
25-
17-
IB: α-HA
IB: α-Flag
IB: α-T7
IB: α-Myc

Fig2 G

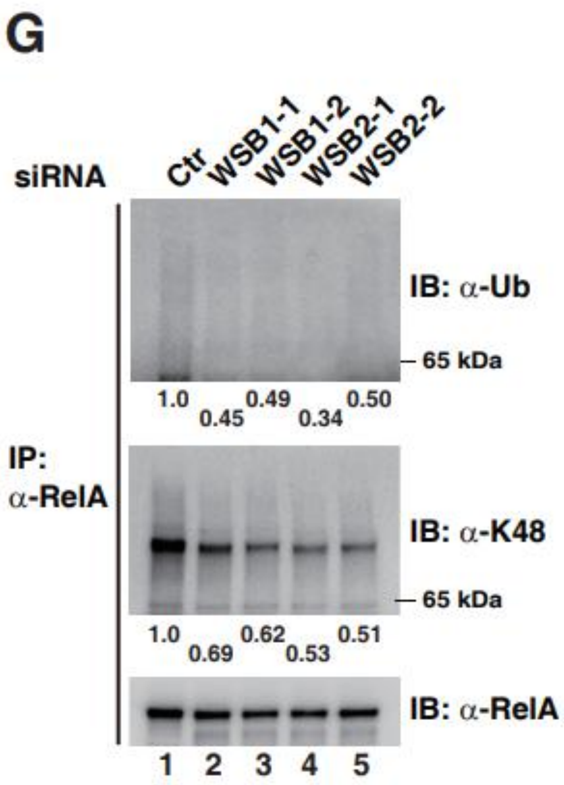
G
siRNA
Ctr
WSB1-1
WSB1-2
WSB2-1
WSB2-2
IP:
α-RelA
IB: α-Ub
65 kDa
1.0
0.45
0.49
0.34
0.50
IB: α-K48
65 kDa
1.0
0.69
0.62
0.53
0.51
IB: α-RelA
1
2
3
4
5

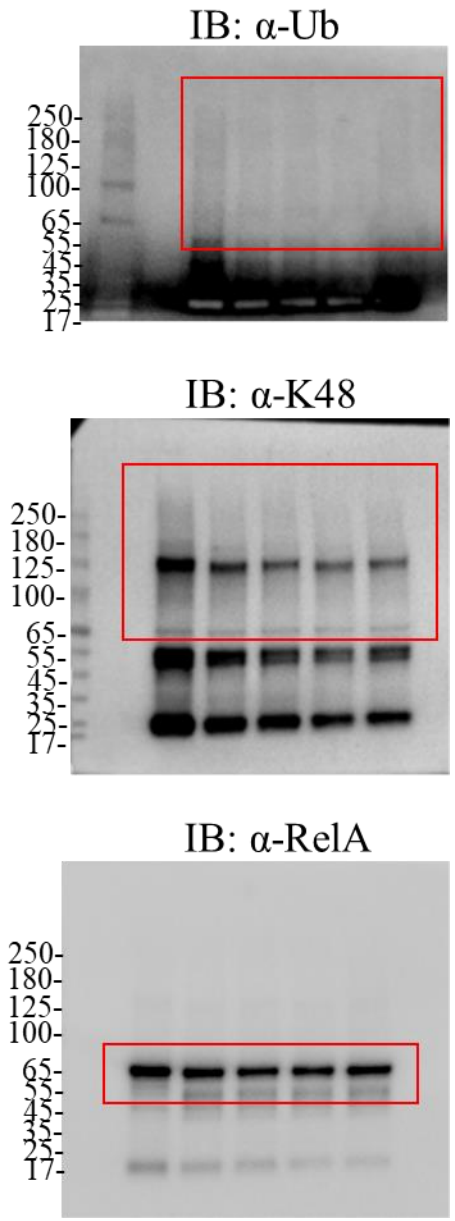
IB: α-Ub
250-
180-
125-
100-
65-
55-
45-
35-
25-
17-
IB: α-K48
250-
180-
125-
100-
65-
55-
45-
35-
25-
17-
IB: α-RelA
250-
180-
125-
100-
65-
55-
45-
35-
25-
17-

Fig3 A

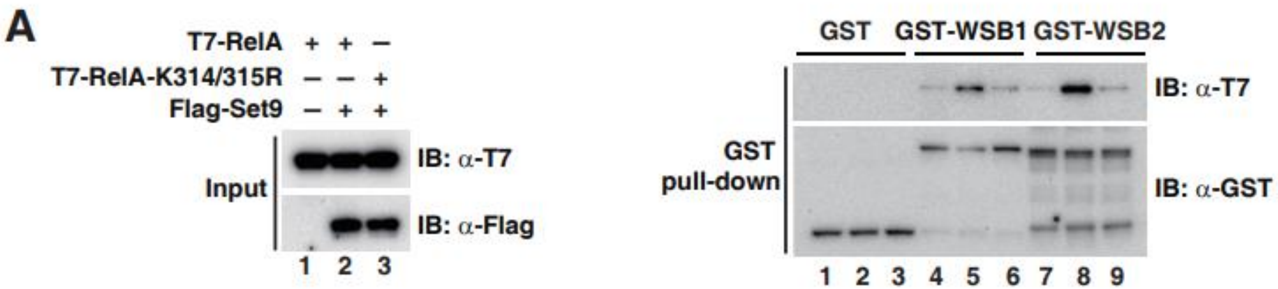
A
T7-RelA + + –
T7-RelA-K314/315R – – +
Flag-Set9 – + +
Input
IB: α-T7
IB: α-Flag
1 2 3
GST GST-WSB1 GST-WSB2
GST pull-down
IB: α-T7
IB: α-GST
1 2 3 4 5 6 7 8 9

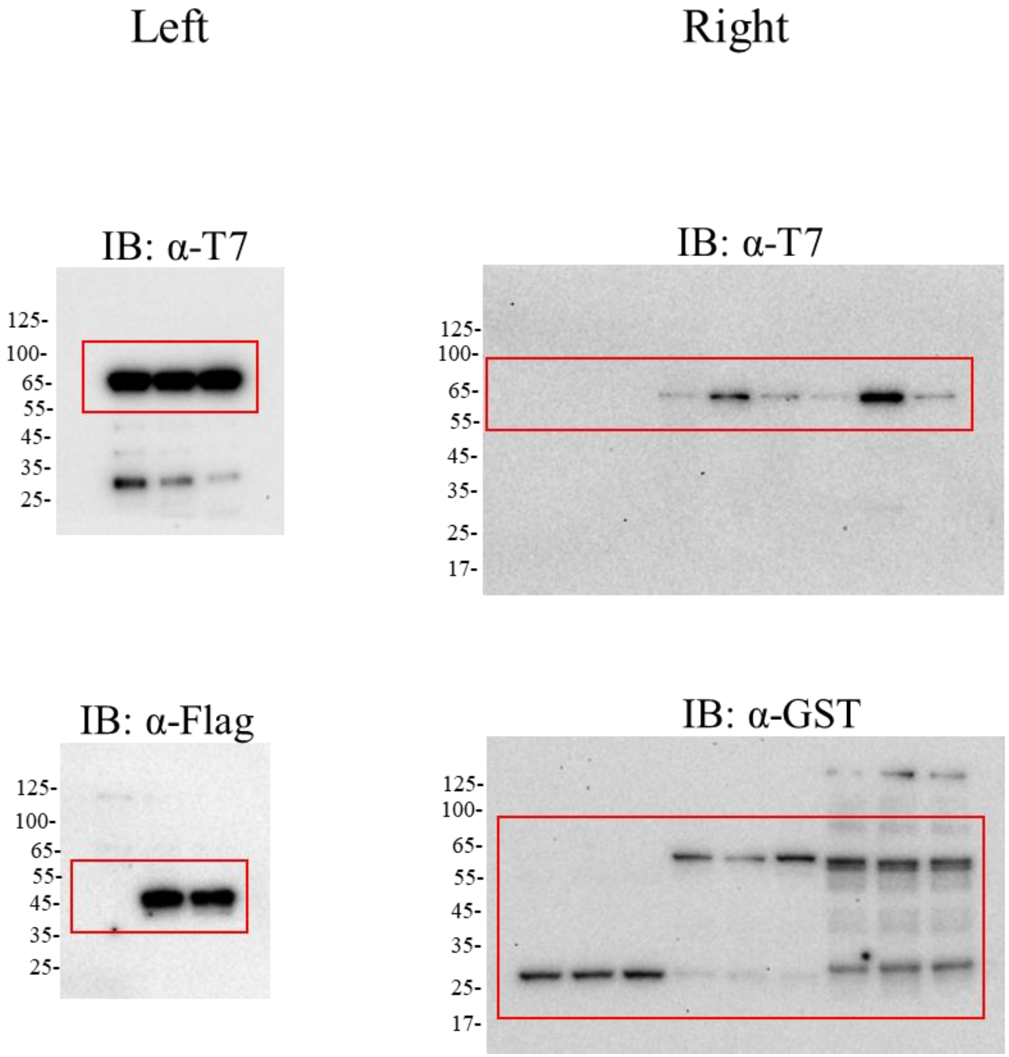
Left
Right
IB: α-T7
125-
100-
65-
55-
45-
35-
25-
IB: α-Flag
IB: α-T7
17-
IB: α-GST

Fig3 B

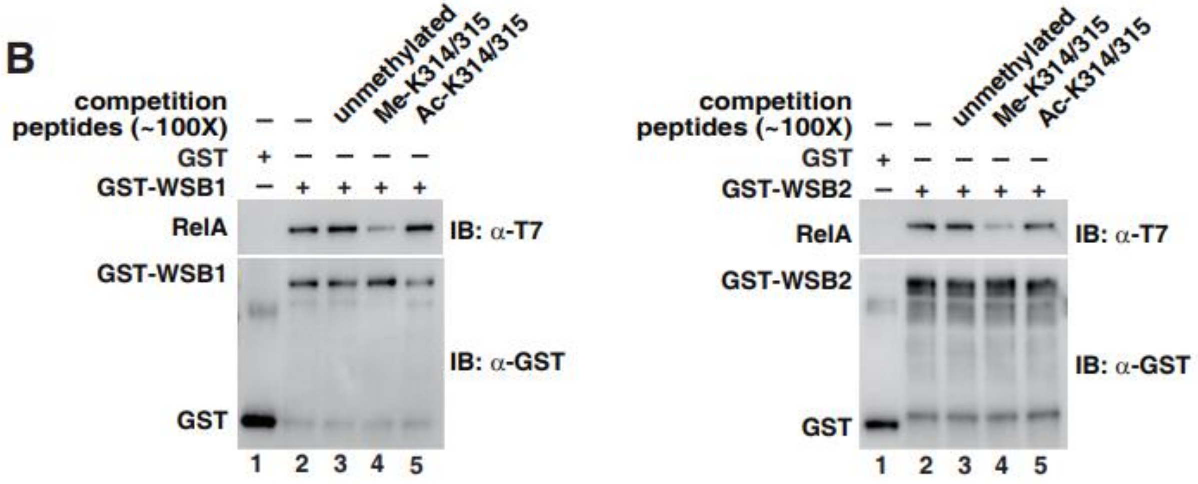
B
competition peptides (~100X)
unmethylated
Me-K314/315
Ac-K314/315
GST
GST-WSB1
RelA
IB: α-T7
GST-WSB1
IB: α-GST
GST
1 2 3 4 5
competition peptides (~100X)
unmethylated
Me-K314/315
Ac-K314/315
GST
GST-WSB2
RelA
IB: α-T7
GST-WSB2
IB: α-GST
GST
1 2 3 4 5

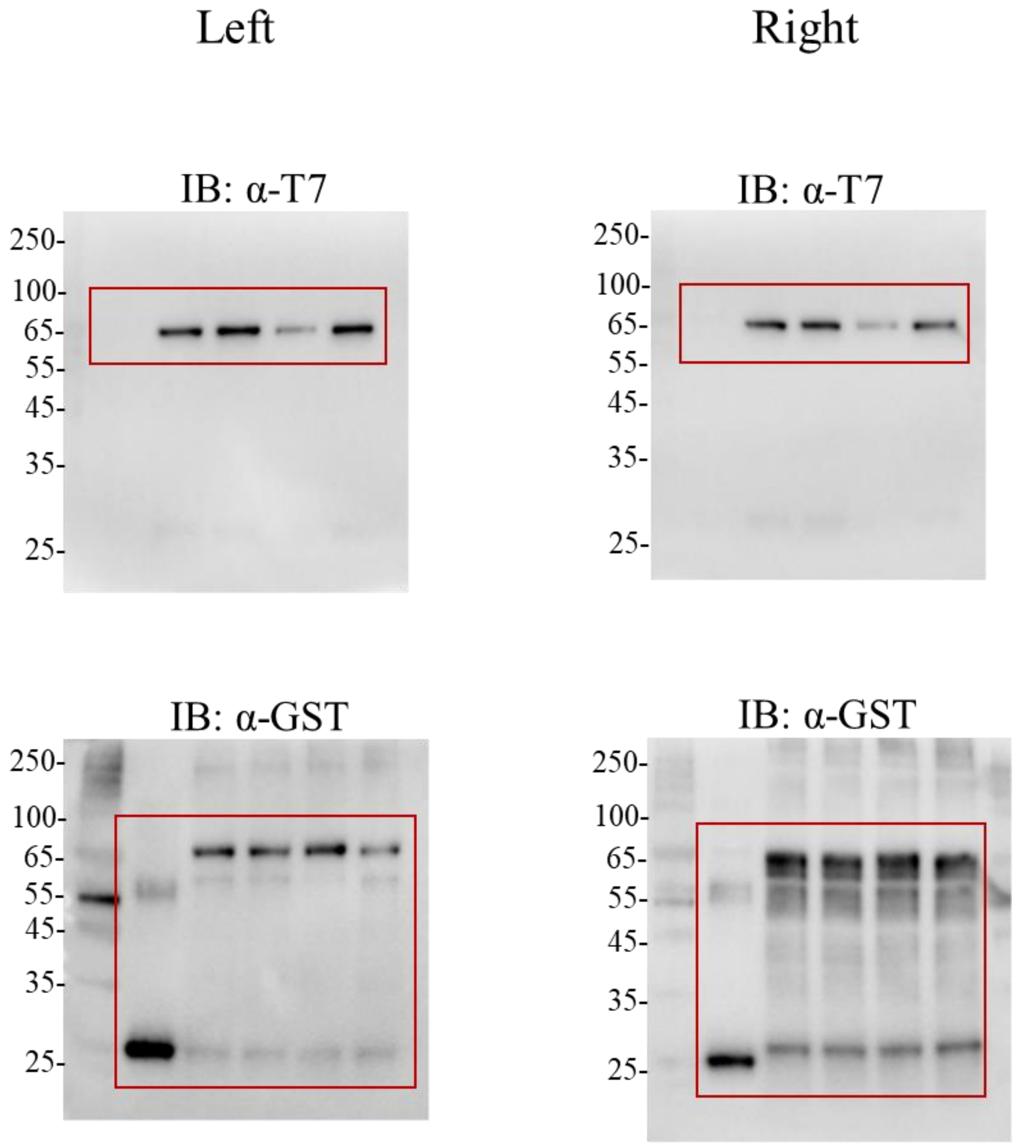
Left
Right
IB: α-T7
250-
100-
65-
55-
45-
35-
25-
IB: α-T7
IB: α-GST
IB: α-GST

Fig3 C

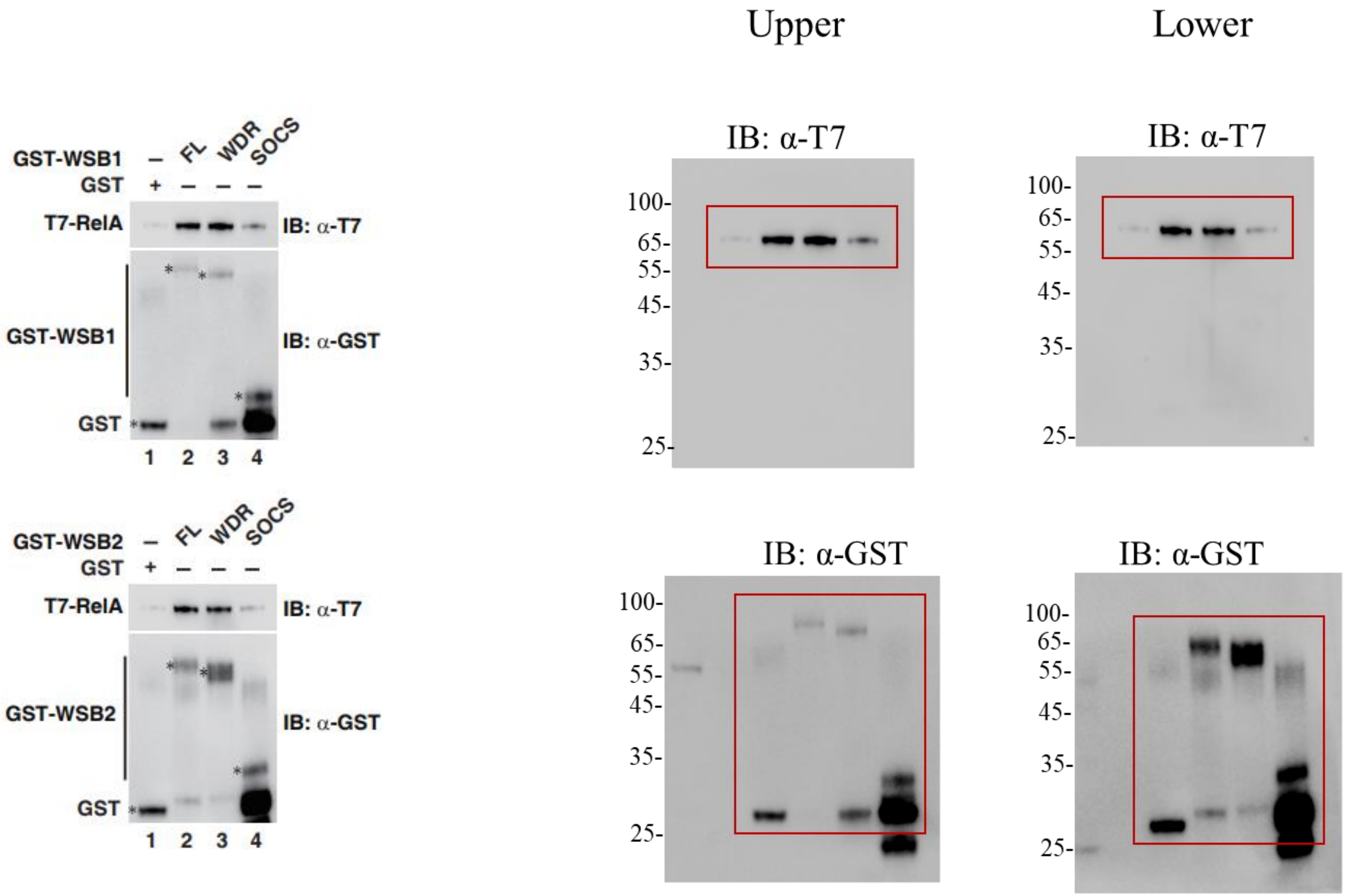
GST-WSB1
GST
— FL WDR SOCS
+ — — —
T7-RelA
IB: α-T7
GST-WSB1
IB: α-GST
GST
1 2 3 4
GST-WSB2
GST
— FL WDR SOCS
+ — — —
T7-RelA
IB: α-T7
GST-WSB2
IB: α-GST
GST
1 2 3 4
Upper
Lower
IB: α-T7
100-
65-
55-
45-
35-
25-
IB: α-T7
100-
65-
55-
45-
35-
25-
IB: α-GST
100-
65-
55-
45-
35-
25-
IB: α-GST
100-
65-
55-
45-
35-
25-

Fig3 D

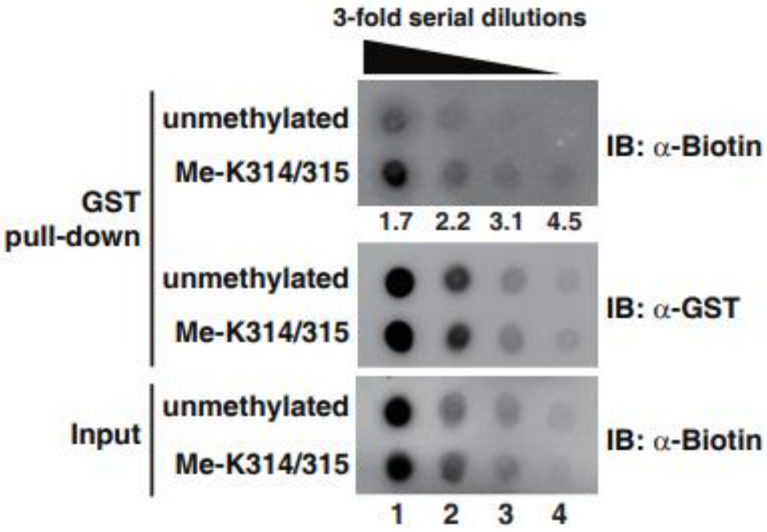
3-fold serial dilutions
GST
pull-down
unmethylated
Me-K314/315
IB: α-Biotin
1.7
2.2
3.1
4.5
unmethylated
Me-K314/315
IB: α-GST
Input
unmethylated
Me-K314/315
IB: α-Biotin
1
2
3
4

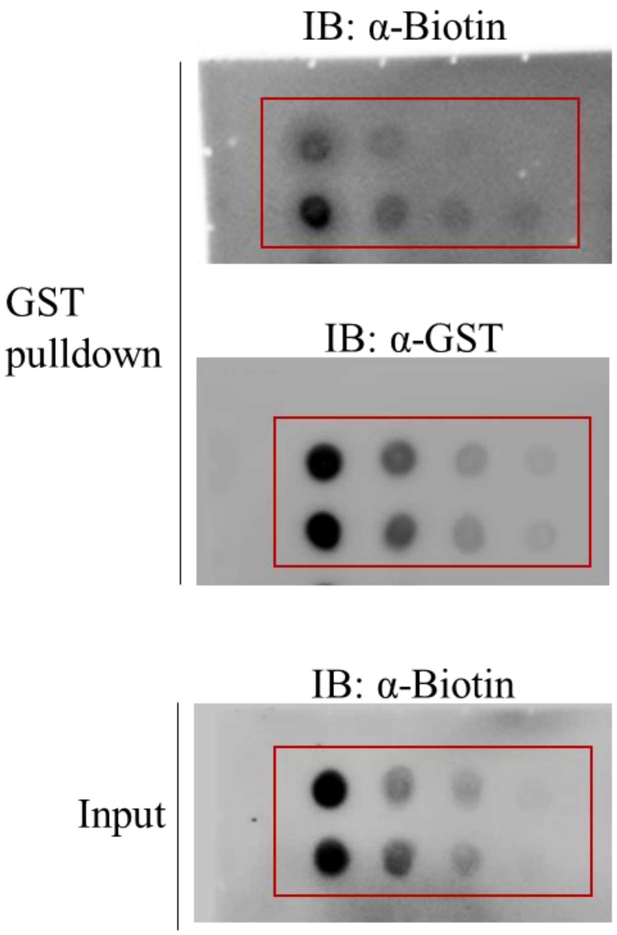
IB: α-Biotin
GST
pulldown
IB: α-GST
IB: α-Biotin
Input

Fig 6 E

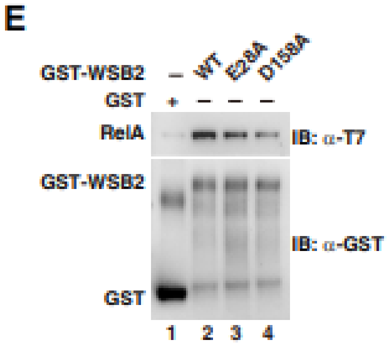
E
GST-WSB2
—
WT
E28A
D158A
GST
+
RelA
IB: α-T7
GST-WSB2
IB: α-GST
GST
1 2 3 4

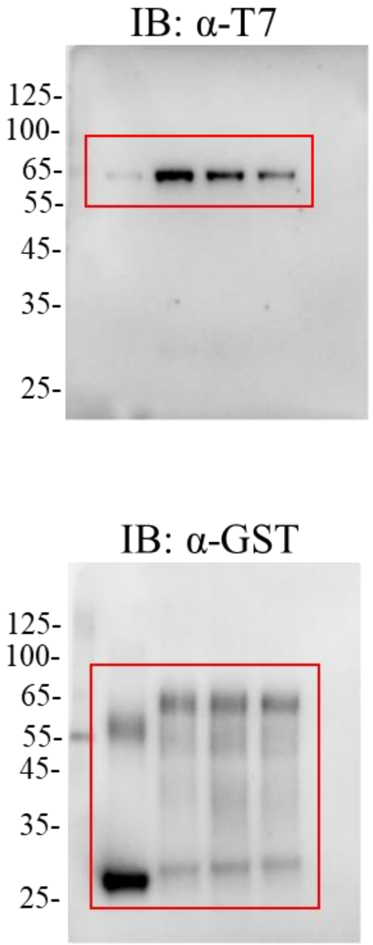
IB: α-T7
125-
100-
65-
55-
45-
35-
25-
IB: α-GST
125-
100-
65-
55-
45-
35-
25-

Fig6 F left

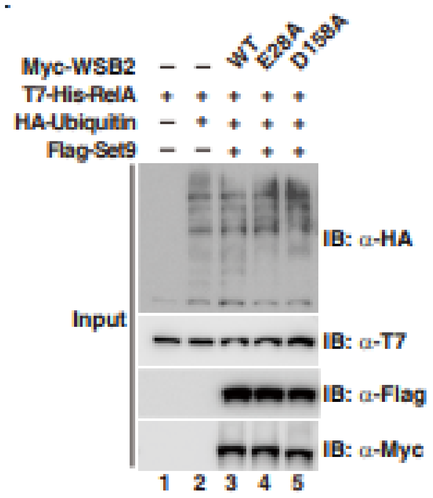
WT
E28A
D158A
Myc-WSB2
T7-His-RelA
HA-Ubiquitin
Flag-Set9
Input
IB: α-HA
IB: α-T7
IB: α-Flag
IB: α-Myc
1 2 3 4 5

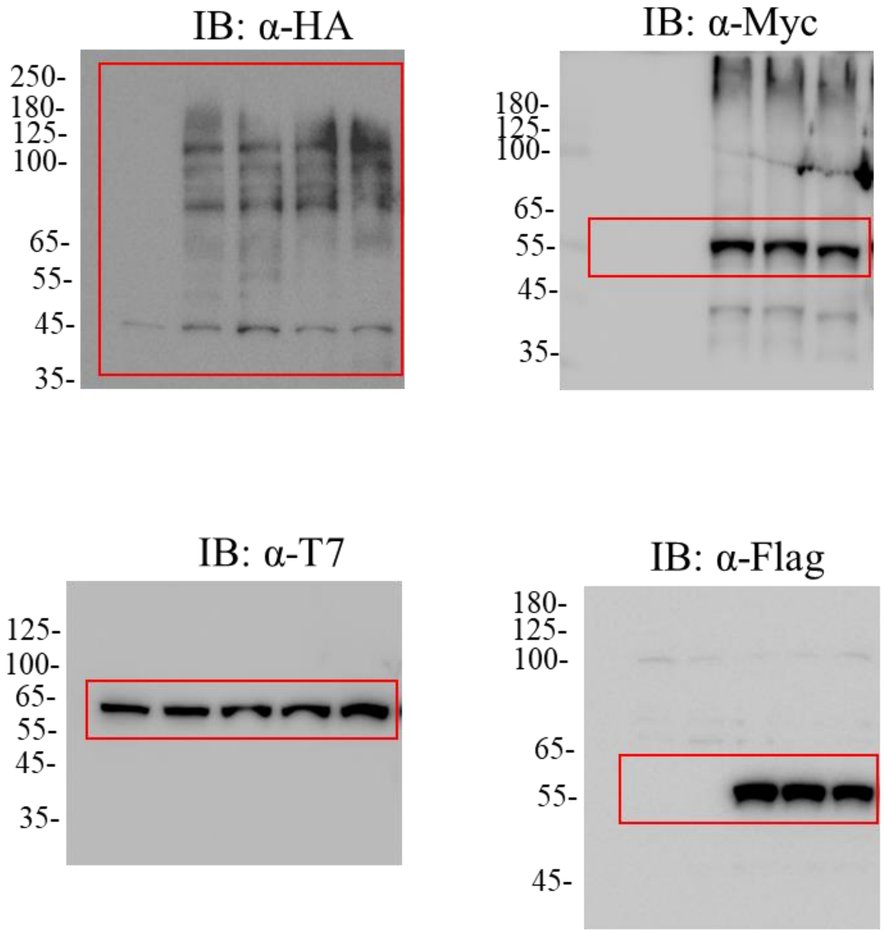
IB: α-HA
250-
180-
125-
100-
65-
55-
45-
35-
IB: α-Myc
180-
125-
100-
65-
55-
45-
35-
IB: α-T7
125-
100-
65-
55-
45-
35-
IB: α-Flag
180-
125-
100-
65-
55-
45-

Fig6 F right

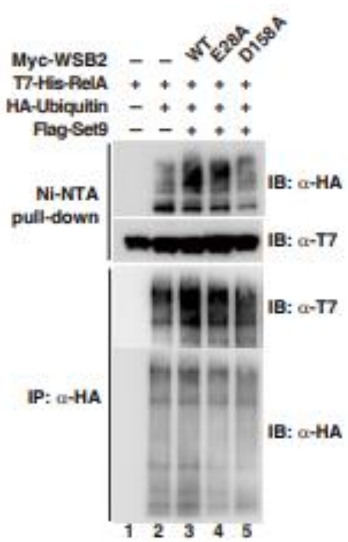

Myc-WSB2
T7-His-RelA
HA-Ubiquitin
Flag-Set9
WT
E28A
D158A
Ni-NTA pull-down
IB: α-HA
IB: α-T7
IP: α-HA
IB: α-T7
IB: α-HA
1 2 3 4 5

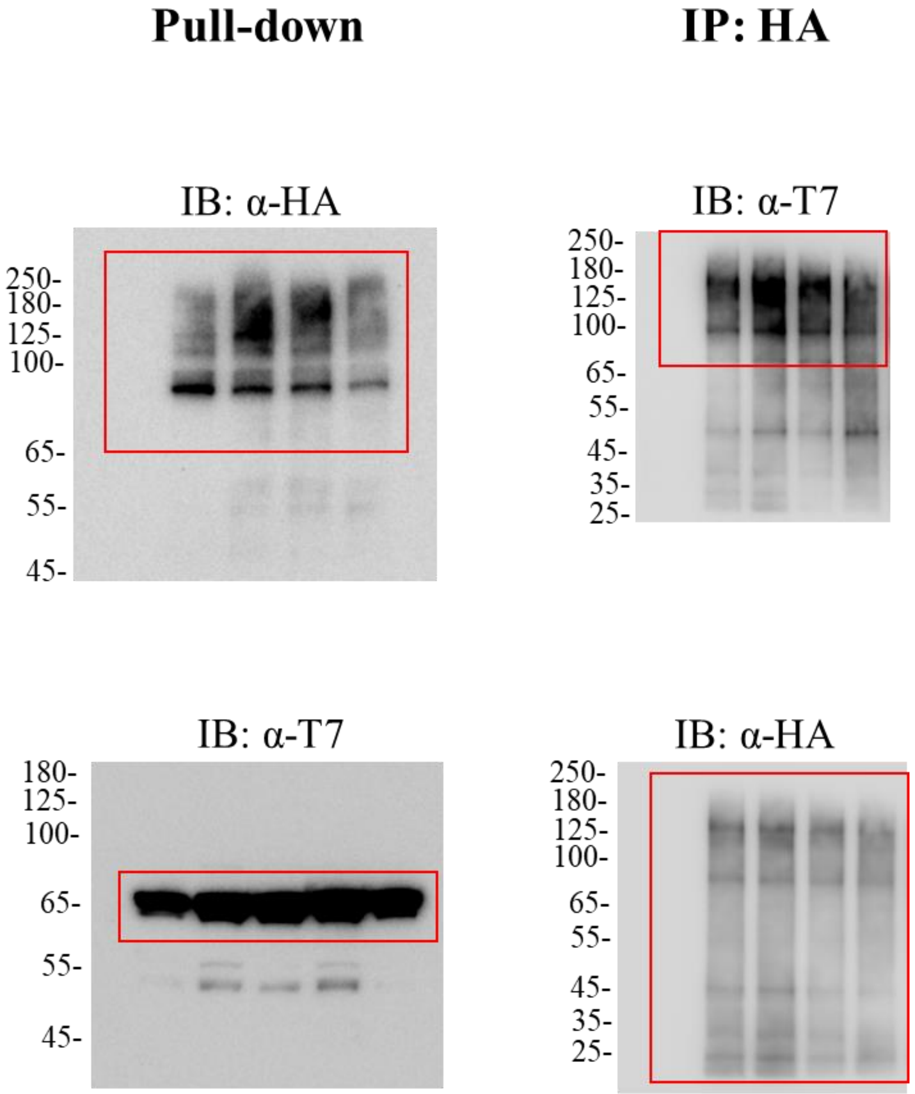

Pull-down
IP: HA
IB: α-HA
250-
180-
125-
100-
65-
55-
45-
IB: α-T7
250-
180-
125-
100-
65-
55-
45-
35-
25-
IB: α-T7
180-
125-
100-
65-
55-
45-
IB: α-HA
250-
180-
125-
100-
65-
55-
45-
35-
25-

Fig S1

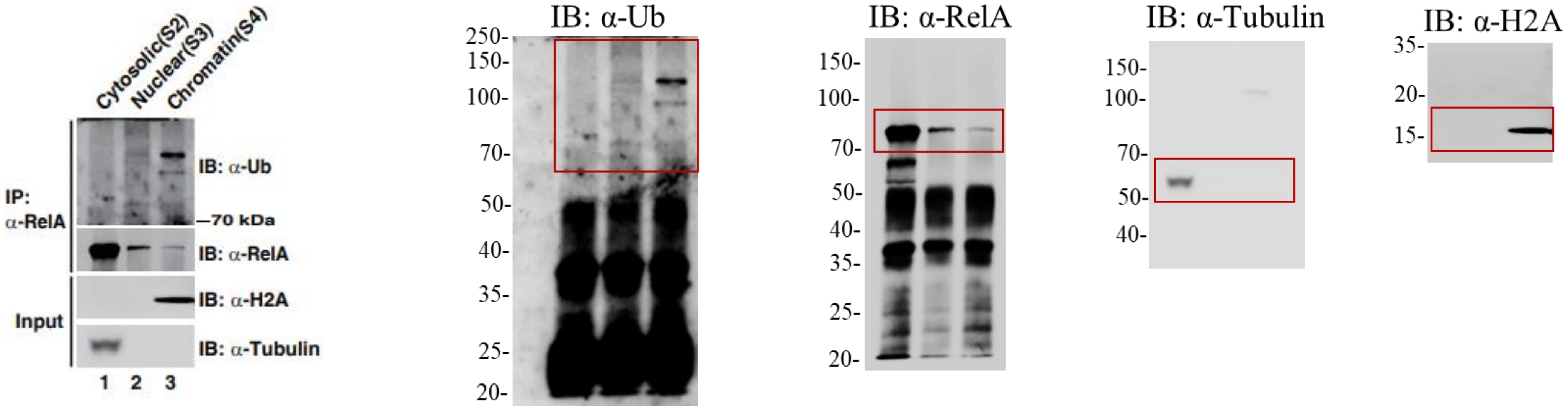

Cytosolic(S2)
Nuclear(S3)
Chromatin(S4)
IP: α-RelA
IB: α-Ub
—70 kDa
IB: α-RelA
Input
IB: α-H2A
IB: α-Tubulin
1 2 3
IB: α-Ub
250-
150-
100-
70-
50-
40-
35-
25-
20-
IB: α-RelA
150-
100-
70-
50-
40-
35-
25-
20-
IB: α-Tubulin
150-
100-
70-
50-
40-
IB: α-H2A
35-
20-
15-

Fig S2

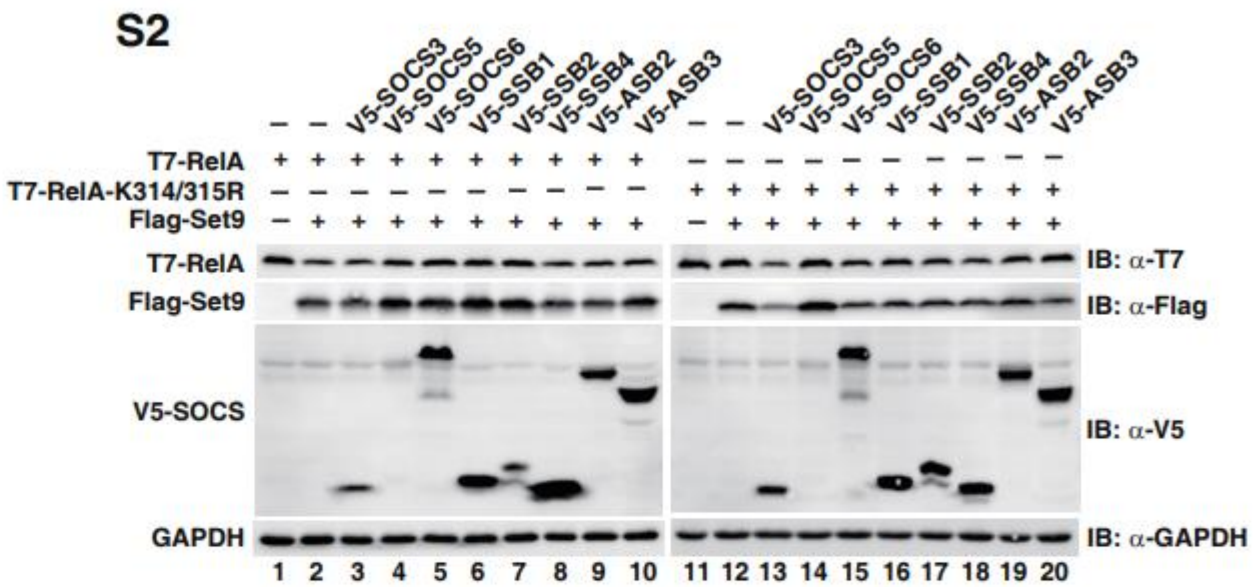
S2
V5-SOCS3
V5-SOCS5
V5-SOCS6
V5-SSB1
V5-SSB2
V5-SSB4
V5-ASB2
V5-ASB3
T7-RelA
T7-RelA-K314/315R
Flag-Set9
T7-RelA
Flag-Set9
V5-SOCS
GAPDH
IB: α-T7
IB: α-Flag
IB: α-V5
IB: α-GAPDH
1 2 3 4 5 6 7 8 9 10 11 12 13 14 15 16 17 18 19 20

Fig S2

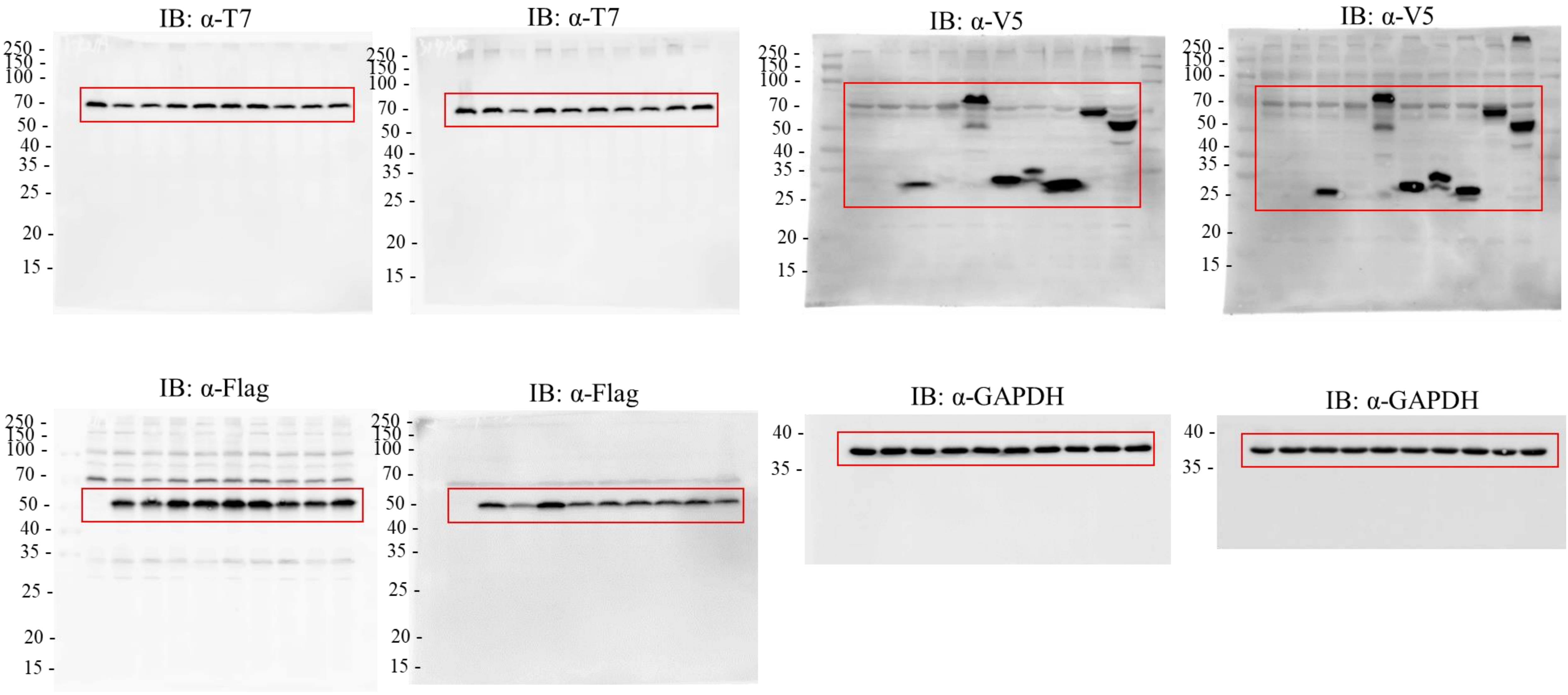
IB: α-T7
250 -
150 -
100 -
70 -
50 -
40 -
35 -
25 -
20 -
15 -
IB: α-T7
IB: α-V5
IB: α-V5
IB: α-Flag
IB: α-Flag
IB: α-GAPDH
40 -
35 -
IB: α-GAPDH

Fig S3 left

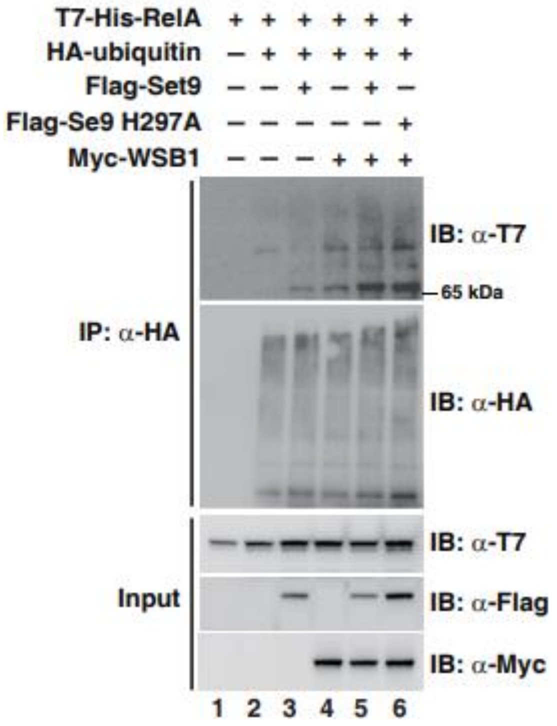

T7-His-RelA + + + + + +
HA-ubiquitin – + + + + +
Flag-Set9 – – + – + –
Flag-Se9 H297A – – – – – +
Myc-WSB1 – – – + + +
IP: α-HA
IB: α-T7
65 kDa
IB: α-HA
Input
IB: α-T7
IB: α-Flag
IB: α-Myc
1 2 3 4 5 6

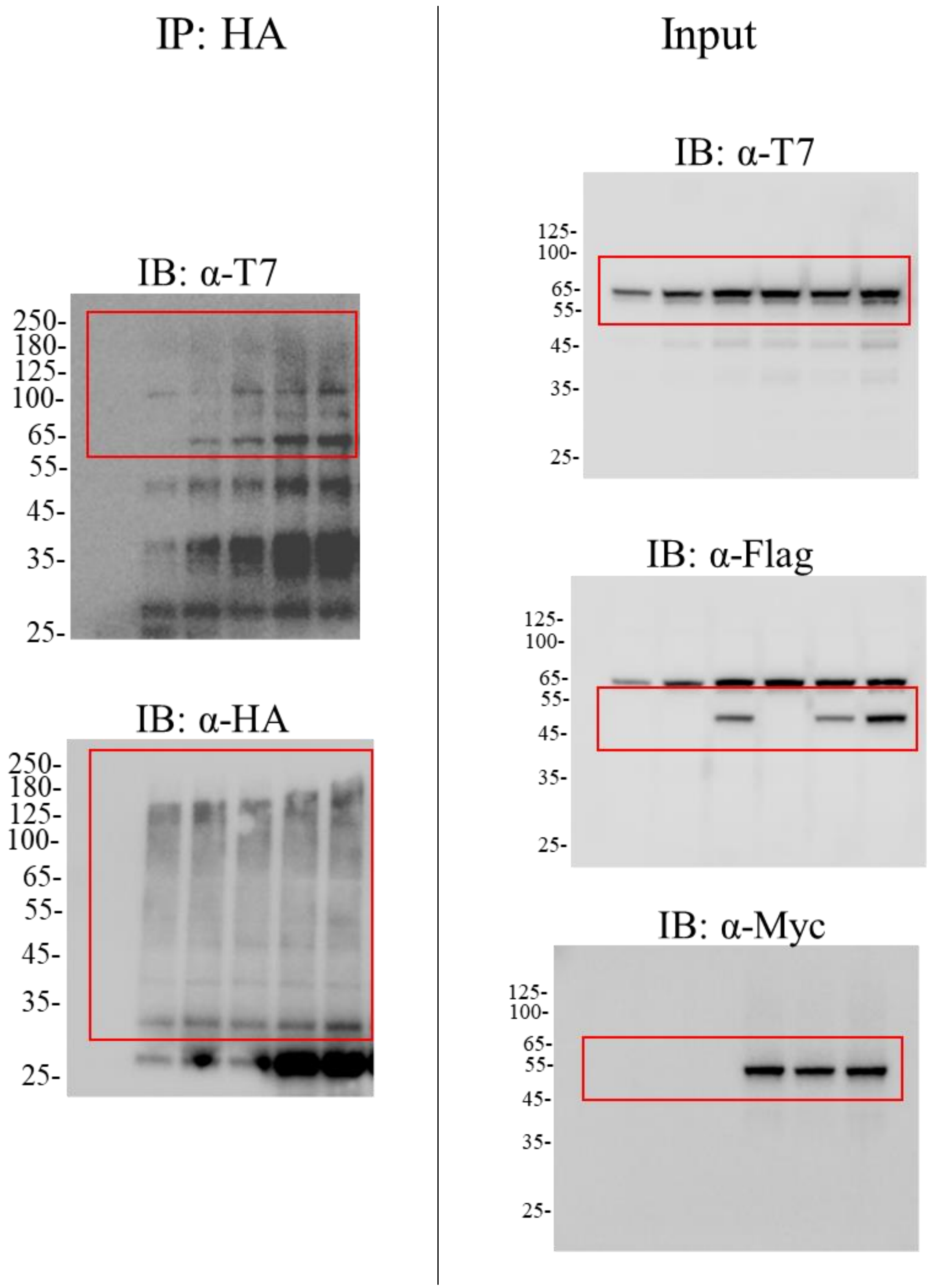

IP: HA
IB: α-T7
250-
180-
125-
100-
65-
55-
45-
35-
25-
IB: α-HA
250-
180-
125-
100-
65-
55-
45-
35-
25-
Input
IB: α-T7
125-
100-
65-
55-
45-
35-
25-
IB: α-Flag
125-
100-
65-
55-
45-
35-
25-
IB: α-Myc
125-
100-
65-
55-
45-
35-
25-

Fig S3 right

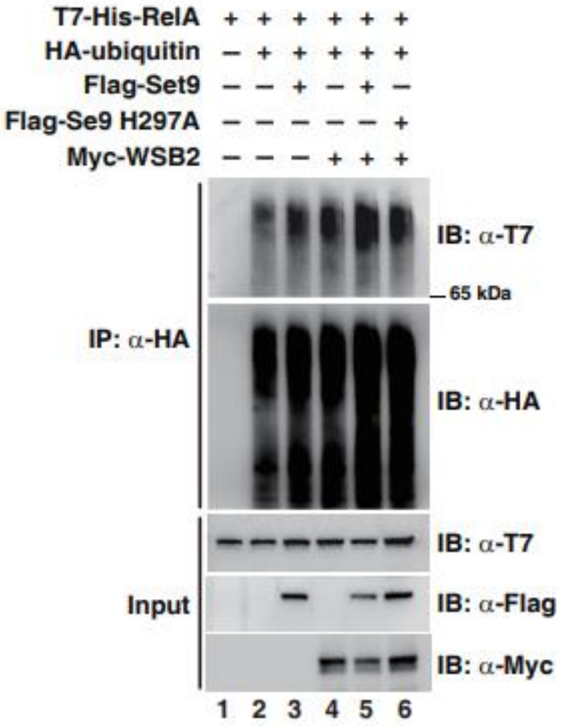

T7-His-RelA + + + + + +
HA-ubiquitin − + + + + +
Flag-Set9 − − + − + −
Flag-Se9 H297A − − − − − +
Myc-WSB2 − − − + + +
IP: α-HA
IB: α-T7
65 kDa
IB: α-HA
Input
IB: α-T7
IB: α-Flag
IB: α-Myc
1 2 3 4 5 6

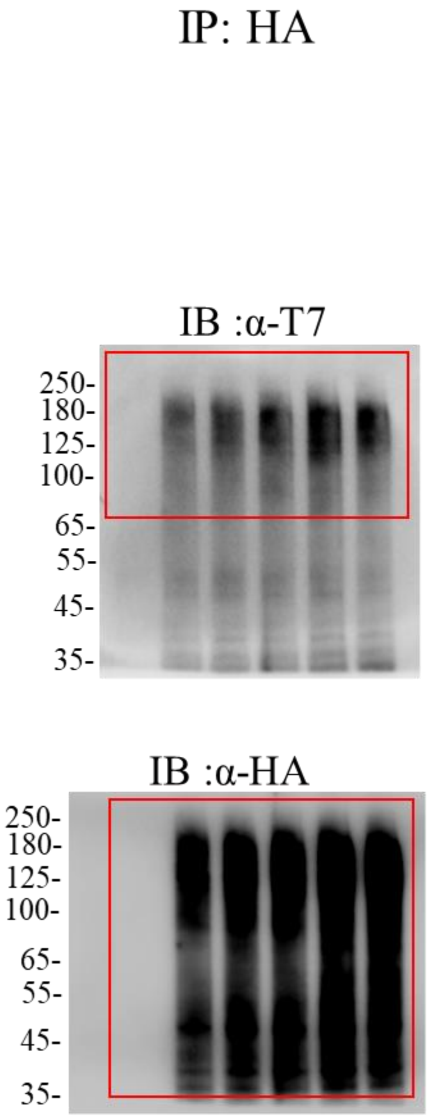

IP: HA
IB :α-T7
250-
180-
125-
100-
65-
55-
45-
35-
IB :α-HA
250-
180-
125-
100-
65-
55-
45-
35-

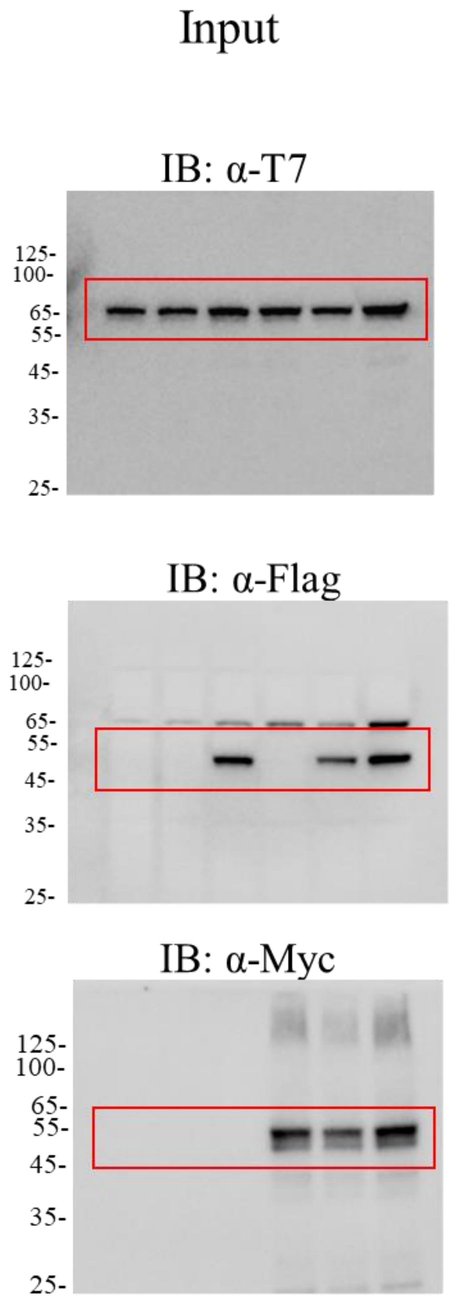

Input
IB: α-T7
125-
100-
65-
55-
45-
35-
25-
IB: α-Flag
125-
100-
65-
55-
45-
35-
25-
IB: α-Myc
125-
100-
65-
55-
45-
35-
25-

Fig S14

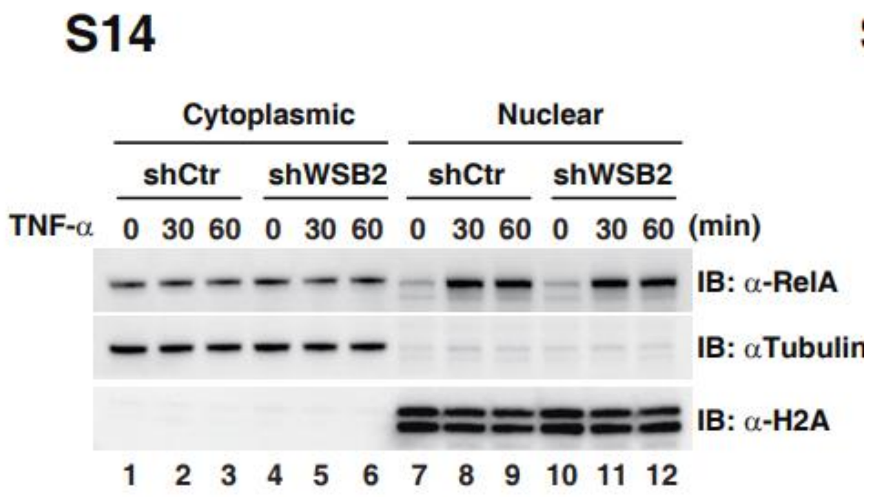
S14
Cytoplasmic
Nuclear
shCtr
shWSB2
shCtr
shWSB2
TNF-α 0 30 60 0 30 60 0 30 60 0 30 60 (min)
IB: α-RelA
IB: αTubulin
IB: α-H2A
1 2 3 4 5 6 7 8 9 10 11 12

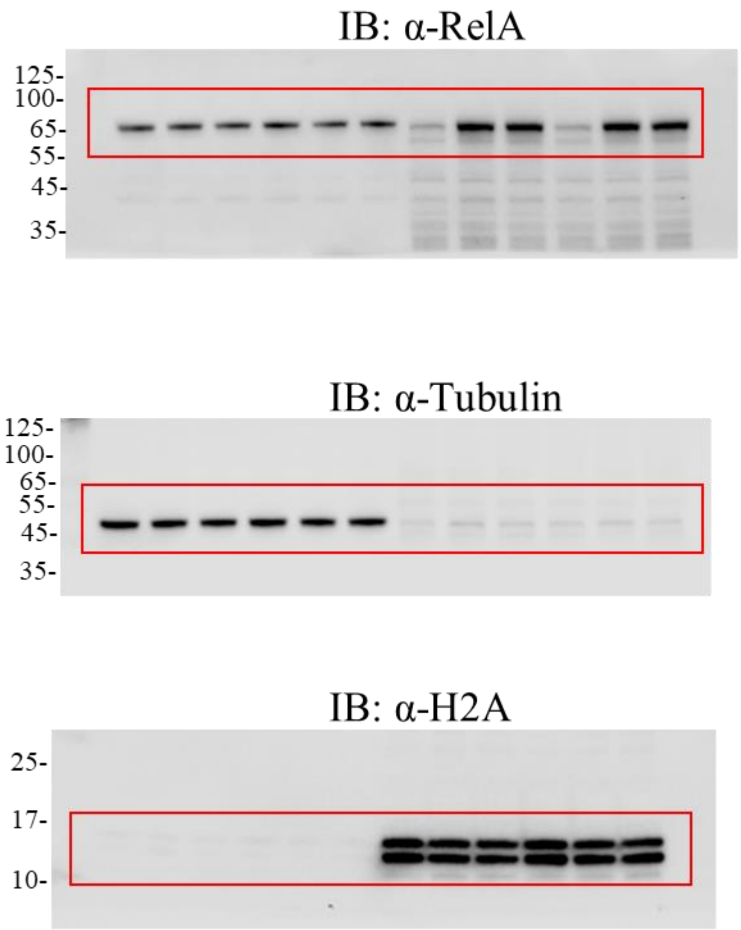
IB: α-RelA
125-
100-
65-
55-
45-
35-
IB: α-Tubulin
125-
100-
65-
55-
45-
35-
IB: α-H2A
25-
17-
10-

Fig S19

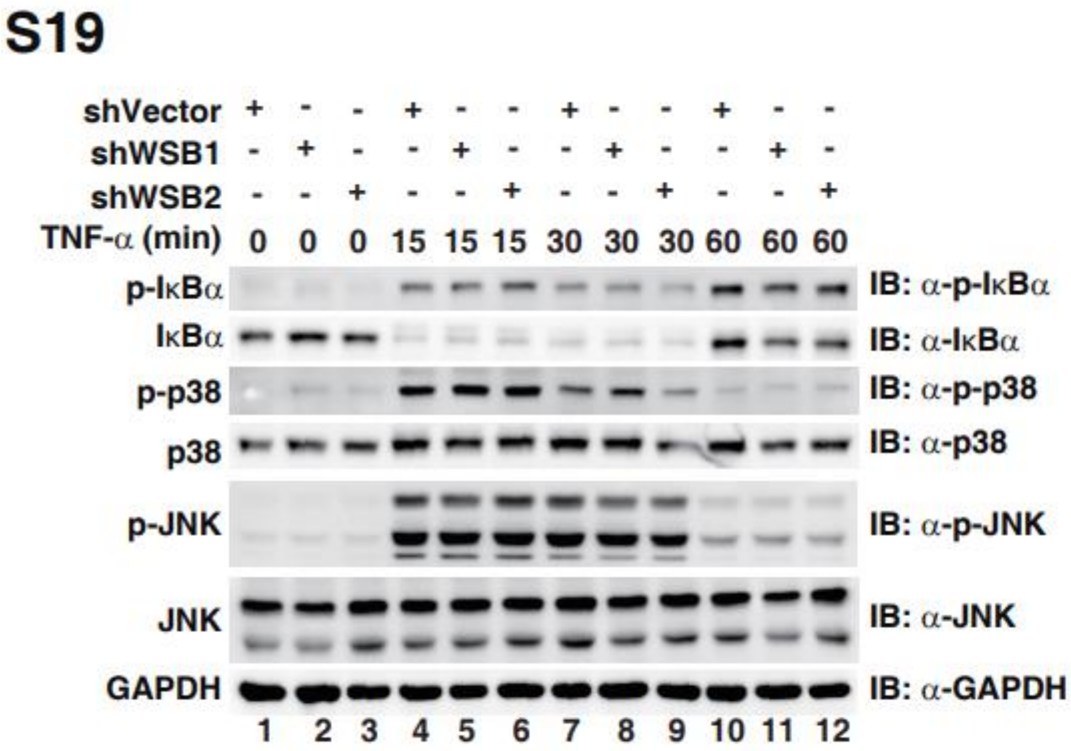

S19
shVector + - - + - - + - - + - -
shWSB1 - + - - + - - + - - + -
shWSB2 - - + - - + - - + - - +
TNF-α (min) 0 0 0 15 15 15 30 30 30 60 60 60
p-IκBα
IκBα
p-p38
p38
p-JNK
JNK
GAPDH
IB: α-p-IκBα
IB: α-IκBα
IB: α-p-p38
IB: α-p38
IB: α-p-JNK
IB: α-JNK
IB: α-GAPDH
1 2 3 4 5 6 7 8 9 10 11 12

Fig S19

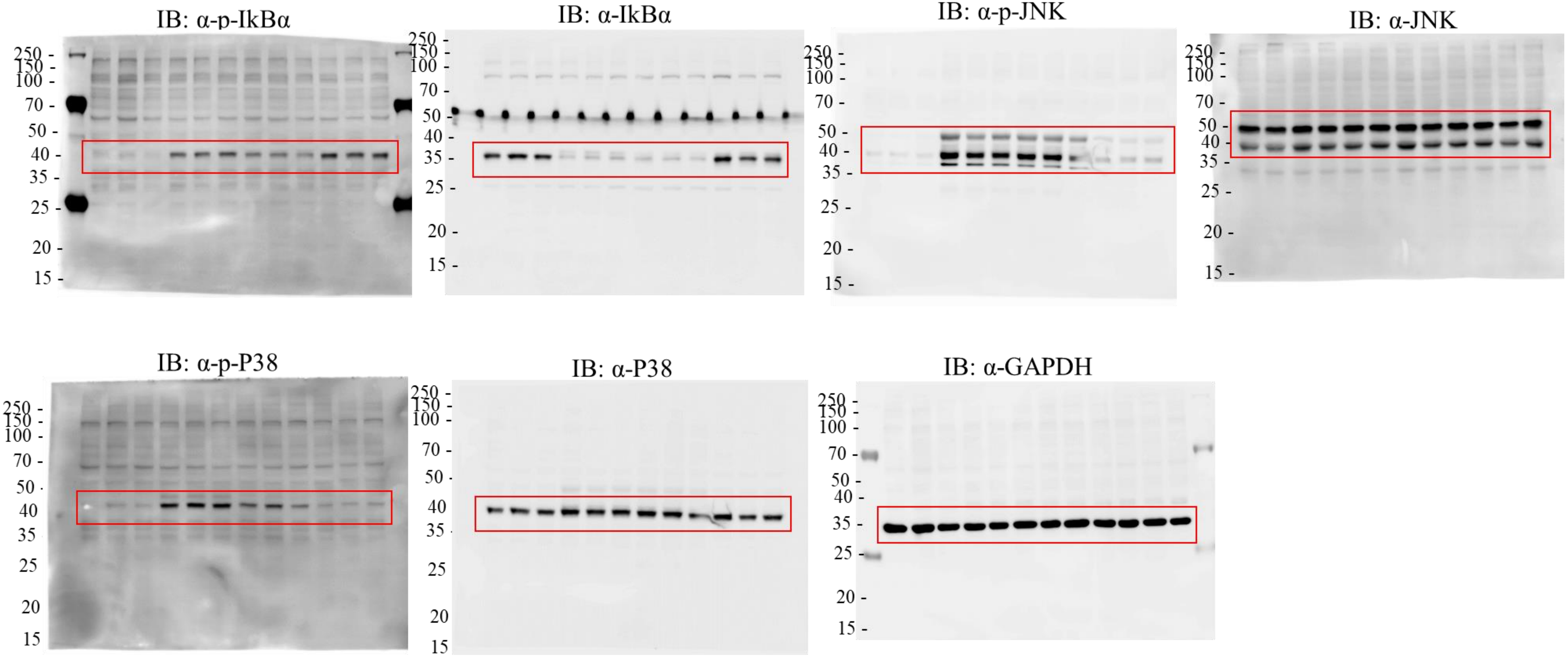
IB: α-p-IkBα
250
150
100
70
50
40
35
25
20
15
IB: α-IkBα
250
150
100
70
50
40
35
25
20
15
IB: α-p-JNK
250
150
100
70
50
40
35
25
20
15
IB: α-JNK
250
150
100
70
50
40
35
25
20
15
IB: α-p-P38
250
150
100
70
50
40
35
25
20
15
IB: α-P38
250
150
100
70
50
40
35
25
20
15
IB: α-GAPDH
250
150
100
70
50
40
35
25
20
15
