# Supplementary material for: WSB1/2 target chromatin-bound lysine-methylated RelA for proteasomal degradation and NF-κB termination
Source: Nucleic Acids Res. 2024 Mar 7;52(9):4969–84. doi: 10.1093/nar/gkae161 (PMC11109945; doi:10.1093/nar/gkae161)
Supplement: gkae161_Supplemental_Files [file gkae161_supplemental_files.zip › Supplementary data-2.pdf]

Supplementary figures

S1

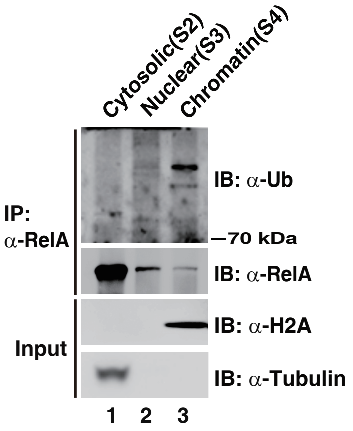

S2

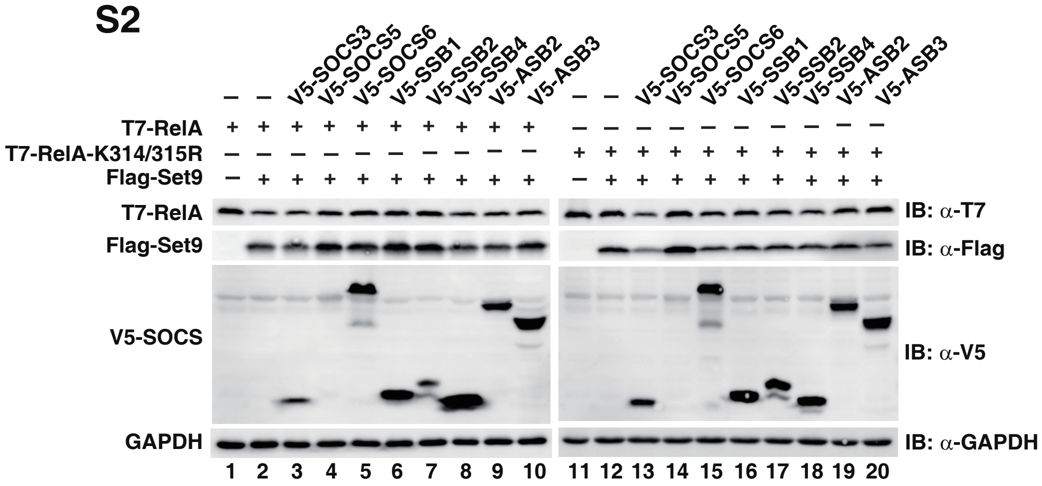

S3

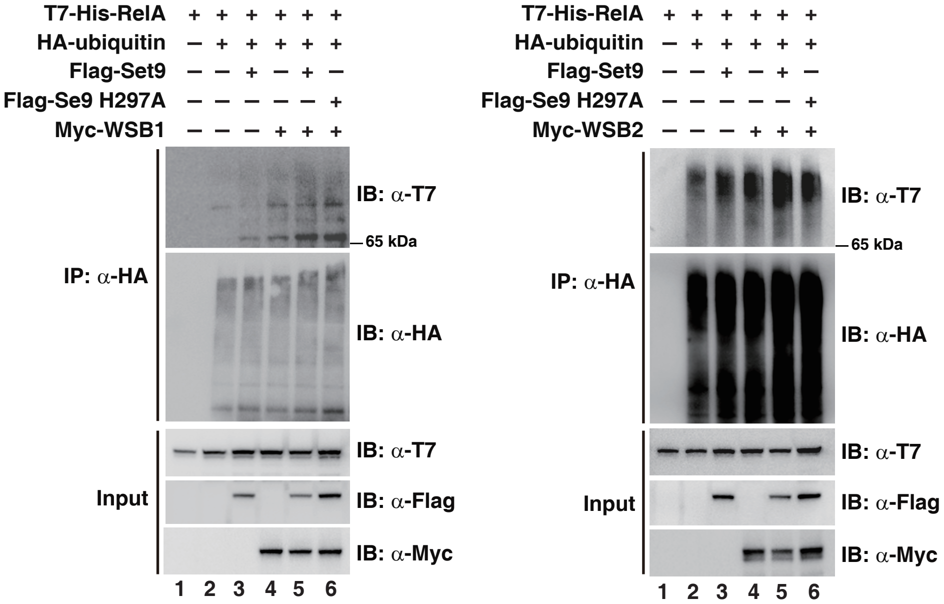

S4

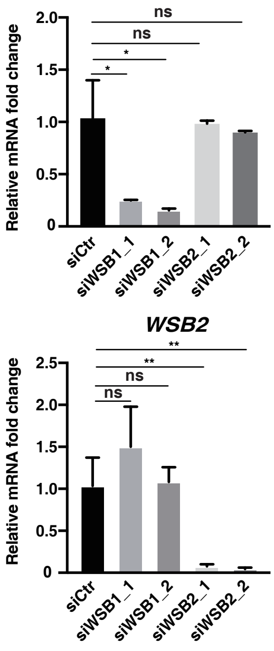

S5

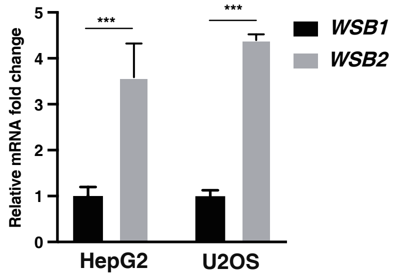

S6

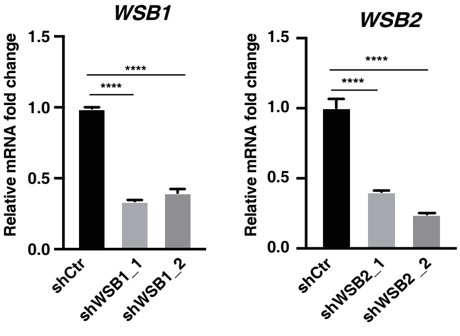

S7

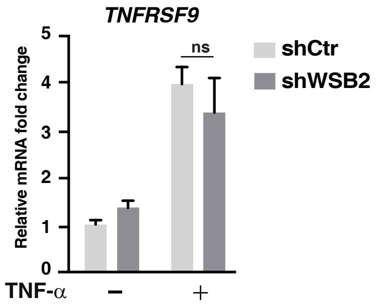

S8

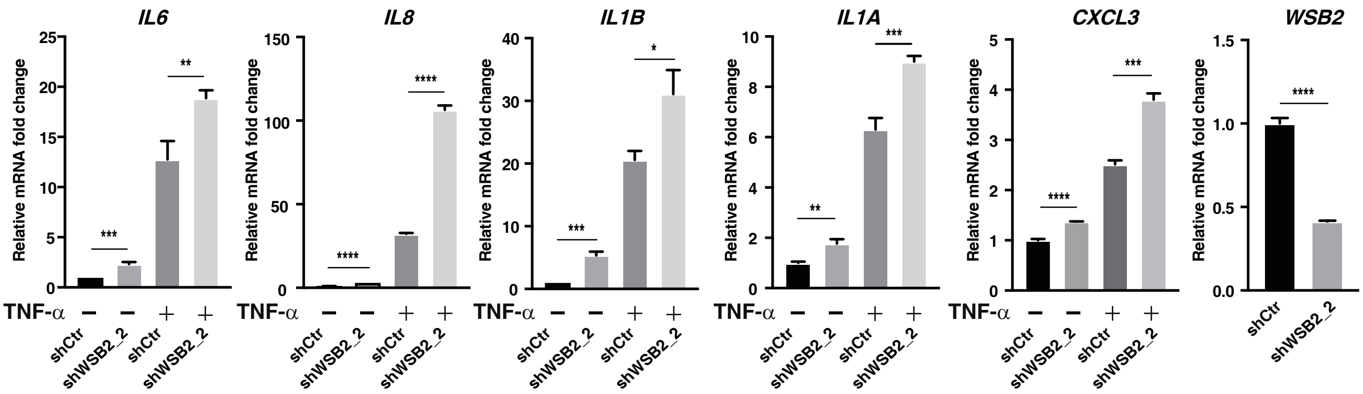

Supplementary figures

S9

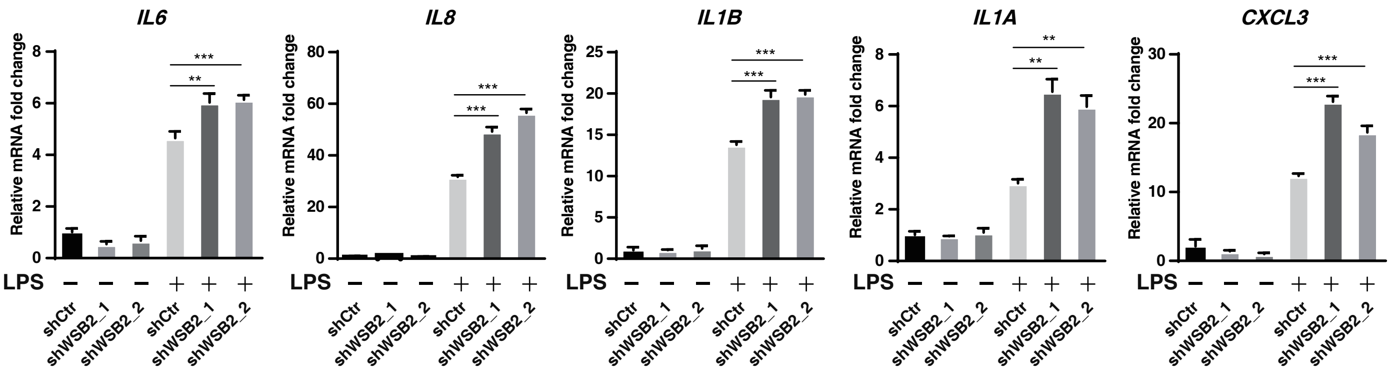

S10

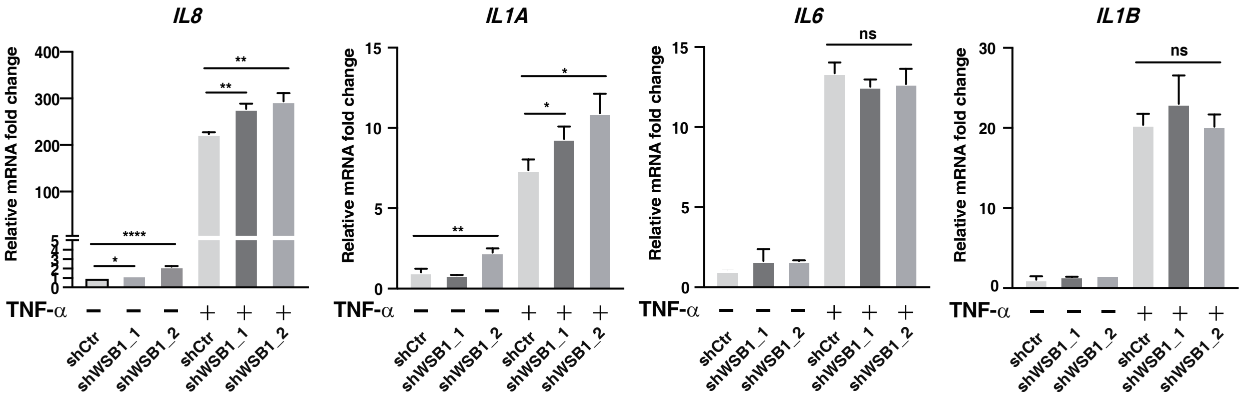

S11

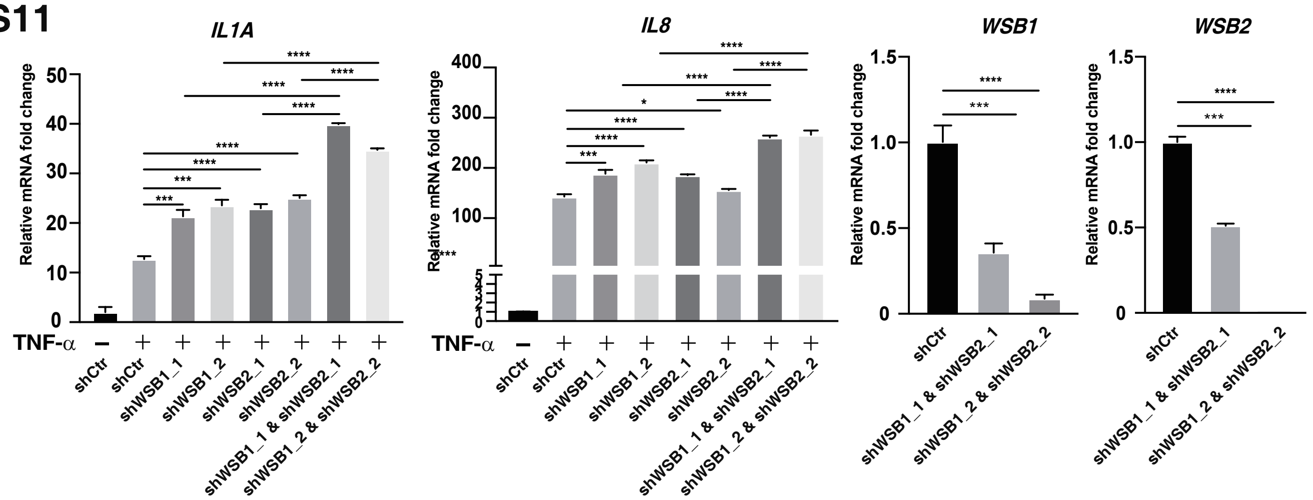

S12

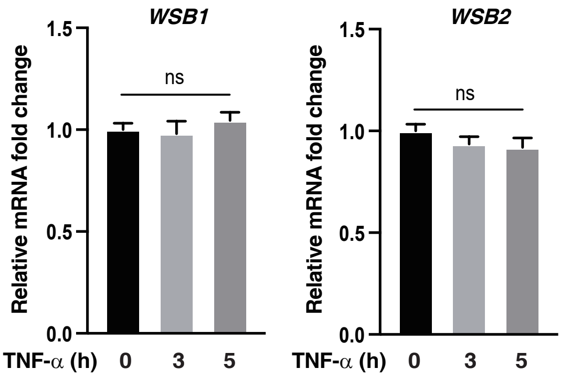

S13

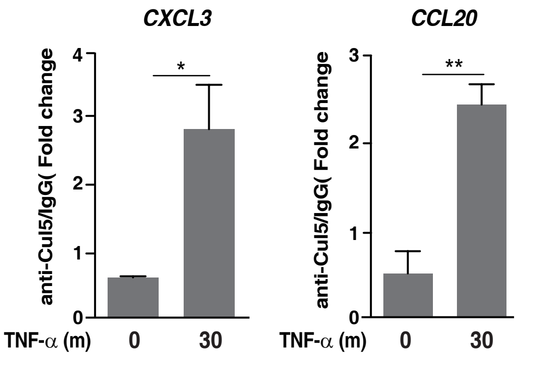

Supplementary figures

S14

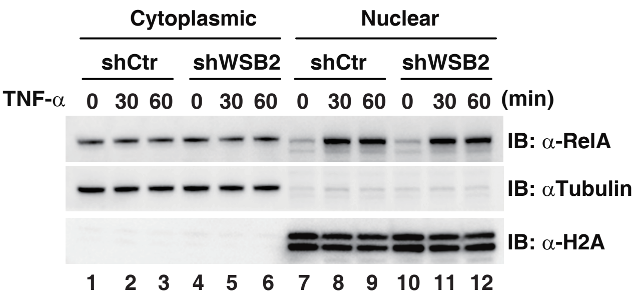

S15

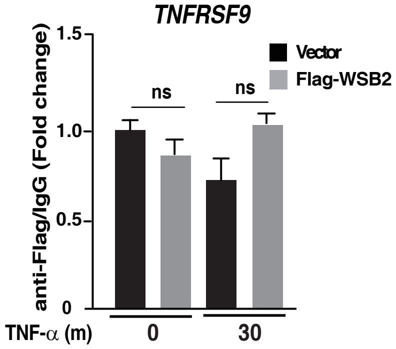

S16

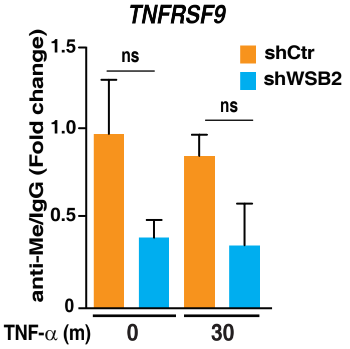

S17

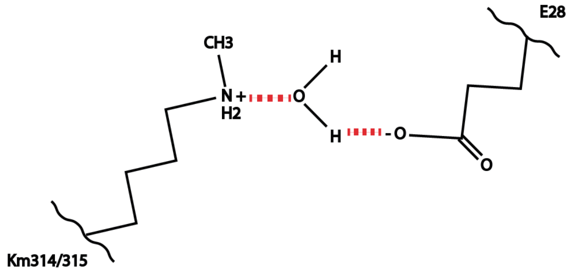

S19

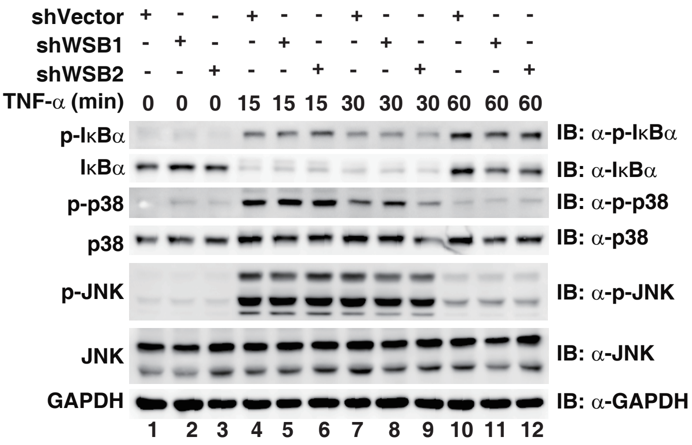

S18

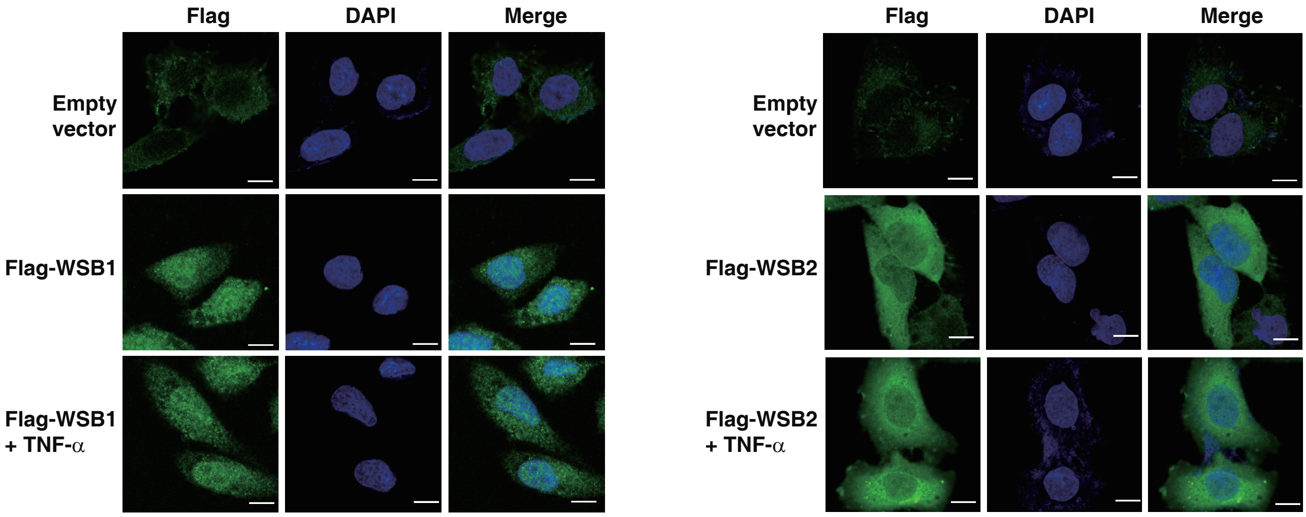

Supplementary figure legends

Figure S1. LPS stimulates ubiquitination of RelA in different fractions. U2OS cells were pulse-stimulated with LPS for 15 min followed by treatment with proteasome inhibitor MG132 for 5 h. The cytoplasmic, nuclear, and chromatin-enriched fractions were prepared and ubiquitination of RelA was assessed as in Figure 1B.

Figure S2. Other E3s from the SOCS family do not degrade RelA or do so in a methylation-independent manner. HEK293T cells were transfected with plasmids encoding T7-tagged WT RelA or RelA-K314/315R and Flag-tagged Set9 together with different V5-tagged SOCS family members as indicated. Whole cell lysates were prepared for IB analysis of all these proteins.

Figure S3. Reciprocal immunoprecipitation showing that WSB1/2 promote RelA ubiquitination in a methylation-dependent manner (related to figure 2 E & 2F). HEK293T cells were transfected with indicated plasmids and treated with 10 μM MG-132 for 5h before harvesting. After lysis under denaturing condition, lysates were used for was immunoprecipitated with anti-HA antibodies and immunoblotted with anti-T7 antibodies for RelA ubiquitination. Levels of tagged RelA, Set9 and WSB1 (left) or WSB2 (left) are shown as input in the lower panels.

Figure S4. Knockdown efficiency of WSB1 and WSB2 by siRNAs. RT-PCR analysis of RNAs extracted from U2OS cells used for the ubiquitination experiment shown in Figure 2G was done to measure the mRNA levels for WSB1 and WSB2. *, $P < 0.05$; **, $P < 0.01$; ns, no significant. Statistical analysis was performed using unpaired two-tailed Student's t-test.

Figure S5. Expression of WSB1 and WSB2 in U2OS and HepG2 cells. RT-PCR analysis of RNAs extracted from untreated HepG2 and U2OS cells was conducted to compare the relative expression levels of WSB1 and WSB2 in each cell line. ***, $P < 0.001$. Statistical analysis was performed using unpaired two-tailed Student's t-test.

Figure S6 Knockdown efficiency of WSB1 and WSB2 by shRNAs. RT-PCR analysis of RNAs extracted from U2OS cells stably expressing empty vector (shCtr) or 2 different shRNAs against WSB1 (shWSB1-1 and shWSB1-2) or WSB2 (shWSB2-1 and shWSB2-2) was conducted to measure the mRNA levels for WSB1 and WSB2. ****, $P < 0.0001$. Statistical analysis was performed using unpaired two-tailed Student's t-test.

Figure S7. Transcription of *TNFRSF9* is not affected by WSB2 knockdown. RT-PCR analysis of RNAs extracted from U2OS cells stably expressing an empty shRNA vector (shCtr) or a shRNA against WSB2 (shWSB2) was performed to measure the relative levels of TNF-α-induced expression of *TNFRSR9*. ns, no significant. Statistical analysis was performed using unpaired two-tailed Student's t-test.

Figure S8. WSB2 down-regulates TNF-α-induced transcription of NF-κB target genes in HepG2 cells. HepG2 cells transduced with a control shRNA (shCtr) or a shRNA against WSB2 (shWSB2) were stimulated with or without TNF-α for 5 h and total RNA was extracted for RT-PCR analysis of NF-κB target genes. *, $P < 0.05$; **, $P < 0.01$; ***, $P < 0.001$; ****, $P < 0.0001$. Statistical analysis was performed using unpaired two-tailed Student's t-test.

Figure S9. WSB2 down-regulates LPS-induced transcription of NF-κB target genes in U2OS cells. U2OS cells transduced with a control shRNA (shCtr) or 2 distinct shRNAs against WSB2 (shWSB2-1 and shWSB2-2) as described in Figure 4D were stimulated with or without LPS for 5 h and total RNA was extracted for RT-PCR analysis of NF-κB target genes. **, $P < 0.01$; ***, $P < 0.001$. Statistical analysis was performed using unpaired two-tailed Student's t-test.

Figure S10. WSB1 down-regulates TNF-α stimulated expression of fewer NF-κB target genes. U2OS cells transduced with a control shRNA (shVector) or 2 distinct shRNAs against WSB1 (shWSB1-1 and shWSB1-2) were stimulated with or without TNF-α for 5 h and total RNA was extracted for RT-PCR analysis of NF-κB target genes. *, $P < 0.05$; **, $P < 0.01$; ***, $P < 0.001$; ****, $P < 0.0001$; ns, no significant. Statistical analysis was performed using unpaired two-tailed Student's t-test.

Figure S11. Double knockdown of WSB1 and WSB2 further enhances transcription of *IL-8* and *IL1A*. RT-PCR analysis of RNAs extracted from U2OS cells stably expressing empty vector (shCtr), a single of 2 different shRNAs against WSB1 (shWSB1-1 and shWSB1-2) or WSB2 (shWSB2-1 and shWSB2-2), or a pair of shRNAs against WSB1 and WSB2, respectively, was done to measure the relative levels of TNF-α-induced expression of *IL-8* and *IL1A* with the level of gene expression in unstimulated shCtr cells set to 1. The knockdown efficiency of *WSB1* and *WSB2* were also determined by RT-PCR and shown to the right. *, $P < 0.05$; **, $P < 0.01$; ***, $P < 0.001$; ****, $P < 0.0001$; ns, no significant. Statistical analysis was performed using unpaired two-tailed Student's t-test.

Figure S12. Constitutive expression of WSB1 and WSB2. U2OS cells were unstimulated or stimulated with TNF-α for indicated time points and total RNA was extracted for RT-PCR analysis of expressions of WSB1 and WSB2. ns, no significant. Statistical analysis was performed using unpaired two-tailed Student's t-test.

Figure S13. Cul5 is recruited to WSB2-regulated genes upon TNF-α stimulation. U2OS cells were unstimulated or stimulated with TNF-α for 30 min. ChIP assays with antibodies against Cul5 were performed and statistically analyzed as in Figure 5**A** for the Cul5 association with promoters of indicated genes. * $P < 0.05$; ** $P < 0.01$. Statistical analysis was performed using unpaired two-tailed Student's t-test.

Figure S14. WSB2 does not seem to regulate levels of cytoplasmic and nuclear RelA during TNF-α stimulation. U2OS cells transduced with a control shRNA (shCtl) or a shRNA against WSB2 (shWSB2) were unstimulated or stimulated with TNF-α for 30 or 60 min and cytoplasmic and nuclear fractions were prepared for IB for RelA, tubulin and histone H2A. Statistical analysis was performed using unpaired two-tailed Student's t-test.

Figure S15. Flag-WSB2 is not recruited to the promoter of *TNFRSF9*. U2OS cells stably expressing vector or Flag-tagged WSB2 were stimulated with TNF-α for indicated time points. A ChIP assay with anti-Flag antibodies was performed and statistically analyzed as in Figure 5**A** for WSB2 binding to the promoter of *TNFRSF9*. ns, no significant. Statistical analysis was performed using unpaired two-tailed Student's t-test.

Figure S16. WSB2 knockdown does not lead to accumulation of methylated RelA at the promoter of *TNFRSF9*. U2OS cells stably expressing control or WSB2 shRNAs were stimulated with TNF-α for indicated time points. A ChIP assay with antibodies against K314/315-methylated RelA was performed and statistically analyzed for association of methylated RelA with promoters of *TNFRSF9* as in Figure 5**C**. ns, no significant. Statistical analysis was performed using unpaired two-tailed Student's t-test.

Figure S17. Potential role of E28 in WSB2 binding to methylated RelA (related to Figure 6D). Molecular image of the water-bridged interaction between monomethylated K314 or K315 and E28 in WSB2.

Figure S18. Subcellular Localization of WSB1/2. U2OS cells stably expressing empty vector, Flag-tagged WSB1 (left) or Flag-tagged WSB2 (right) were unstimulated or stimulated with TNF-α for 30 min. Cells were fixed for immunofluorescence with anti-Flag antibodies and staining with a DNA probe DAPI followed by confocal microscopy. Scale bars in each panel represent 50 μm.

Figure S19. Depletion of WSB1 and WSB2 has little effect on TNF-α stimulated upstream signaling events of the NF-κB pathway. U2OS cells transduced with a control shRNA (shCtr) or a shRNAs against WSB1 (shWSB1) or WSB2 (shWSB2) were stimulated with or without TNF-α for indicated time points and protein extracts were prepared for immunoblotting of the indicated proteins.

Table S1. oligo sequences used in this study

	Oligo name	Sequence (5'>3')
siRNA sequences (sense)	Negative control	UUCUCCGAACGUGUCACGUTT
	WSB1 siRNA_1	GAGUCGCUGUGUAAAUAUATT
	WSB1 siRNA_2	GGAGAAUUGAUGAGGAUUATT
	WSB2 siRNA_1	CCCACCAGUUUGAUUGGAATT
	WSB2 siRNA_2	CAUCCCUAAAGGGUUUGAATT
shRNA sequences (antisense)	*WSB1* shRNA_1	AAATACGATACGAGAGAAAC
	WSB1 shRNA_2	ATAATTGAAGTCAGTAAAGC
	WSB2 shRNA_1	TTGGTATCGTAAGAAGCCGTG
	WSB2 shRNA_2	ACTTCCAATCAAACTGGTGGG
Primers for RT-PCR	*GAPDH*_Forward	ATCTCTGCCCCCTCTGCTGA
	*GAPDH*_Reverse	GATGACCTTGCCCACAGCCT
	*CXCL3*_Forward	CGCCCAAACCGAAGTCATAG
	*CXCL3*_Reverse	GCTCCCCTTGTTCAGTATCTTTT
	*IL6*_Forward	ACTCACCTCTTCAGAACGAATTG
	*IL6*_Reverse	CCATCTTTGGAAGGTTCAGGTTG
	*IL8*_Forward	ACTGAGAGTGATTGAGAGTGGAC
	*IL8*_Reverse	AACCCTCTGCACCCAGTTTTC
	*IL1A*_Forward	TGGTAGTAGCAACCAACGGGA
	*IL1A*_Reverse	ACTTTGATTGAGGGCGTCATTC
	*IL1B*_Forward	ATGATGGCTTATTACAGTGGCA
	*IL1B*_Reverse	GTCGGAGATTCGTAGCTGGA
	*CCL20*_Forward	TGCTGTACCAAGAGTTTGCTC
	*CCL20*_Reverse	CGCACACAGACAACTTTTTCTTT
	*TNFRSF9*_Forward	AGCTGTTACAACATAGTAGCCAC
	*TNFRSF9*_Reverse	GGACAGGGACTGCAAATCTGAT
	*WSB1*_Forward	TGTGCCTTCTCTACTGATGGC
	*WSB1*_Reverse	GTGGAGTGGCCCAAAAATACA
	*WSB2*_Forward	TCAAGGACACTGCATCGTCAA
	*WSB2*_Reverse	GGCTTTTGGCTTCAAACCCTTTA
Primers for ChIP	*IL1A*_Forward	CCTCAAGTGATTGTCCTGCCTC
	*IL1A*_Reverse	TTGGGTTCCCAGTTGGAGTT
	*IL1B*_Forward	GGCTGGGCTTCTTCCAAATC
	*IL1B*_Reverse	CCTACGTGTTGGGTGGCAAG
	*IL6*_Forward	ACCCTCACCCTCCAACAAAG
	*IL6*_ Reverse	GCAGAATGAGCCTCAGACATC
	*CXCL3*_Forward	CCTACCCGTATCCGACTCCAC
	*CXCL3*_Reverse	CCAAGATCGGCGAACCCTTT
	*CCL20*_Forward	CCTTCGCACCTTCCCAATATGA
	*CCL20*_Reverse	GAATGTACACAGAAGGCGTGTTG
	*TNFRSF9*_Forward	CTCTAGGGGATTTCGGGGTC
	*TNFRSF9*_Reverse	GGGAAATTCCCACCACAGCT
